# Morphological, functional, and phylogenetic aspects of the head capsule of the cockroach *Ergaula capucina* (Insecta/Blattodea)

**DOI:** 10.7717/peerj.12470

**Published:** 2022-04-19

**Authors:** Benjamin Wipfler, Felix Triesch, Dominic Evangelista, Tom Weihmann

**Affiliations:** 1 Morphologielabor, Zoologisches Forschungsmuseum Alexander Koenig, Bonn, Germany; 2 Institut für Zoologie und Evolutionsforschung mit Phyletischem Museum, Ernst-Haeckel-Haus und Biologiedidaktik, Friedrich-Schiller Universität Jena, Jena, Germany; 3 Biology Department, Adelphi University, Garden City, New York, United States of America; 4 Department of Animal Physiology, University of Cologne, Cologne, Germany

**Keywords:** Morphology, Evolution, Dictyoptera, Polyneoptera, Mechanical advantage, Effective cross section, Biting apparatus, Musculature, Corydiidae

## Abstract

**Background:**

Cockroaches are usually typical omnivorous detritivores and their cephalic morphology is considered to be ancestral in various aspects. Thus, several studies addressed the morphology and function of the blattodean head, and the cockroach usually serves as a model for standard mouthparts in text books. However, so far only two of the three major lineages of Blattodea have been studied and no detailed information for the head of any Corydioidea was available. The present study closes this gap by providing a detailed morphological description of the head of *Ergaula capucina*, studying some important functional parameters of the mandible and discussing it in a phylogenetic framework.

**Methods:**

The cephalic morphology of *Ergaula* studied in detail using a broad set of different techniques including digital microscopy, µ-computed tomography, and 3-dimensional reconstructions. Concerning the functional morphology of the mandible, we compared the volume and effective cross sections of the eight compartments of the primary mandibular adductor muscle for *Ergaula*, *Blattella germanica*, and *Salganea rossi* and measured the mechanical advantage, *i.e.*, the force transmission ratio for all teeth of the mandible of *Ergaula*.

**Results:**

The head capsule of *Ergaula* is characterized by a strong sexual dimorphism and typical orthopteran mouthparts. It resembles the head capsule of other roaches in several respects and confirms oesotendons, the reduction of the mesal occelus, and bipartite M. verticopharyngealis and M. hypopharyngosalivaris as blattodean apomorphies. But it also shows some unique adaptations. It is the first described cockroach that lacks the dorsal tentorial arms which has various consequences for the cephalic musculature. On the maxillary lacinia, *Ergaula* is the first described blattodean to show strong and blunt setae instead of a lacinula, which might be homologues to the dentisetae of dragonflies and mayflies. Like other corydiid roaches that inhabit xeric areas, *Ergaula* has an atmospheric water-vapor absorption mechanism that includes a gland and a ductus on the epipharnyx and bladders on the hypopharynx. The mandibular adductor is in cockroaches asymmetric, a pattern not found in termites, mantids, or other closely related insects.

## Introduction

Cockroaches comprise ~4,600 species (Beccaloni & Eggleton, 2011) that cover a broad variety of different diets. They are generally best described as omnivorous detritivores but cover a wide range of food specialists (Bell, Roth & Nalepa, 2007) and are closely related to the ancestrally wood-feeding termites and the predatory mantises (Abe, Bignell & Higashi, 2000; Prete et al., 1999; Wipfler et al., 2019). The phylogeny of the extant blattodean lineages is discussed in detail in Evangelista et al. (2019).

These moderately diverse insects play an important ecological role and comprise some of the best-known pest species. Yet, the phenotypic correlates of their extant biodiversity (Djernæs, 2018) and ecological niche adaptations (Evangelista, Bourne & Ware, 2014; Hopkins, 2014; Legendre et al., 2014) are largely unexplored. Large-scale morphological studies of cockroaches have either focused on morphological characters with poorly documented functional significance (Klass & Meier, 2006; McKittrick, 1964) or describe structures having an unfortunate combination of plesiomorphic and, apparently, highly derived character states (Rehn, 1951). While the former is appropriate for phylogenetic analysis (Klass & Meier, 2006) and the latter may be useful for taxonomy (*i.e*., DNA barcoding; Hajibabaei et al., 2007), detailed comparative anatomy of structures demonstrating direct behavioral or ecological relevance are rare (*e.g*., see Klass, 1998; Schmidt et al., 2009; van Casteren & Codd, 2010).

One of the best-studied body parts of cockroaches is the head with its appendages. The species that is best studied in this respect is *Periplaneta americana* (Crampton, 1917; Crampton, 1921; Crampton, 1923; Crampton, 1925; Dorsey, 1943; Popham, 1961; Roberts, 1972; Snodgrass, 1960; Walker, 1931; Wipfler et al., 2016) but other studies also dealt with *Blaberus giganteus* (Pradl, 1971), *Blattella germanica* (Pradl, 1971; Snodgrass, 1943; Snodgrass, 1944), *Blatta orientalis* (Miall & Denny, 1886; Pradl, 1971; Strenger, 1939; Yuasa, 1920), and *Parcoblatta pennsylvanica* (Gangwere, 1965). Additionally several studies compared the morphology of the head capsule or specific mouthparts for several cockroach species (Buder & Klass, 2013; Klass & Eulitz, 2007; Mangan, 1908; Zhuzhikov, 2007). The mechanical and functional aspects of the mouthparts of *Periplaneta americana* were addressed by Popham (1961), Roberts (1972), Schmitt, Rack & Betz (2014) and Weihmann et al. (2015a, 2015b).

In the present contribution, we provide a detailed documentation of the head of an evolutionarily and phenotypically distinct species, *Ergaula capucina*. This species belongs to the Corydiidae, a group for which head morphology was not yet studied and that shares a common ancestor with the other cockroaches approximately 130 million years ago (Evangelista et al., 2019). Corydiid cockroaches are known to possess adaptations for surviving in xeric habitats (Bell, Roth & Nalepa, 2007; Evangelista et al., 2019), whereas *Periplaneta americana*, *Blatta orientalis*, and *Blattella germanica* are extreme generalists (Roth & Willis, 1960). *Ergaula capucina* also differs from *Periplaneta* and *Blattella* in that it exhibits a great sexual dimorphism. In order to facilitate a comparative framework and assess phylogenetic implications, we place additional emphasis on the difference between the male and the female head capsule, and specific functional aspects of the mandibular apparatus such as the mechanical advantage.

## Materials and methods

### Animals

The present work is based on adult male and female specimens of *Ergaula capucina* (Brunner of Wattenwyl, 1893) (Polyphagidae/Corydiidae) which were fixed in 70% ethanol. The animals were acquired from the breeding of Jörg Bernhardt (www.schaben-spinnen.de).

### Digital microscopy

Specimens were dissected in 70% ethanol under a stereo microscope (Zeiss Stemi 2000). In order to show mouthparts free of attaching tissue, some samples were macerated with 5% KOH for 2 h at 50 °C. Afterwards, all samples were dried with hexamethyldisilazan (HDMS) (Brown, 1993). Digital microscopy was performed with a Keyence VHX 2000. Image plates were assembled with Adobe Photoshop CS6 and Adobe Illustrator CS6.

### µ-computed tomography (µCT) and 3D reconstructions

The head of a male *Ergaula capucina* was dried with HMDS (see above) and mounted to a sample holder. SR-µ-CT was performed at BESSY2 of the Berliner Elektronenspeicherring-Gesellschaft für Synchrotronstrahlung (Berlin, Germany) with the following parameters: scan dimensions: 4,007 × 4,007 pixels; spatial resolution: 2.486 μm; exposure time: 340 ms; 2,000 projections with an angle of 0.09°. The acquired image stack was segmented with Visage Imaging Amira 5.2.2 and all segmented structures individually exported as tiff-stacks with the algorithm function of Amira. Volume rendering was performed with VG Studiomax 2.2. Image plates were assembled with Adobe Photoshop CS6 and Adobe Illustrator CS6.

### Morphometrics

All morphometric measures were taken from the 3D data provided by the μ-CT scans with Visage Imaging Amira 5.2.2. We measured the inner and outer lever for all teeth of the mandible, the volume (in mm^3^ and % of the complete volume) and effective cross sectional area (in mm^2^ and % of the complete cross sectional area) of the eight compartments of the primary mandibular adductor muscles (M. craniomandibularis internus). Details concerning these factors and their specific measurements are provided in Weihmann et al. (2015a). For comparison, we also measured the effective cross section area and volume of the mandibular adductor for the cockroaches *Blattella germanica, Periplaneta americana*, and *Salganea rossi*. The raw data for the three studied specimens of *Periplaneta americana* is provided in Dataset S1.

### Terminology

The general morphological terminology follows Beutel et al. (2014). Muscles were named and homologized according to Wipfler et al. (2011). If a muscle present in the terminology of Wipfler et al. (2011) is not mentioned, it could not be found in the examination. We used the terminology of Buder & Klass (2013) for hypopharyngeal elements and Wipfler et al. (2016) for cephalic lines. As cockroaches have a highly moveable head, we define it for descriptive and illustrative terms as being orthognathous (*i.e.*, the mouthparts point ventrally).

## Results

### Head capsule

**Figure 1:**
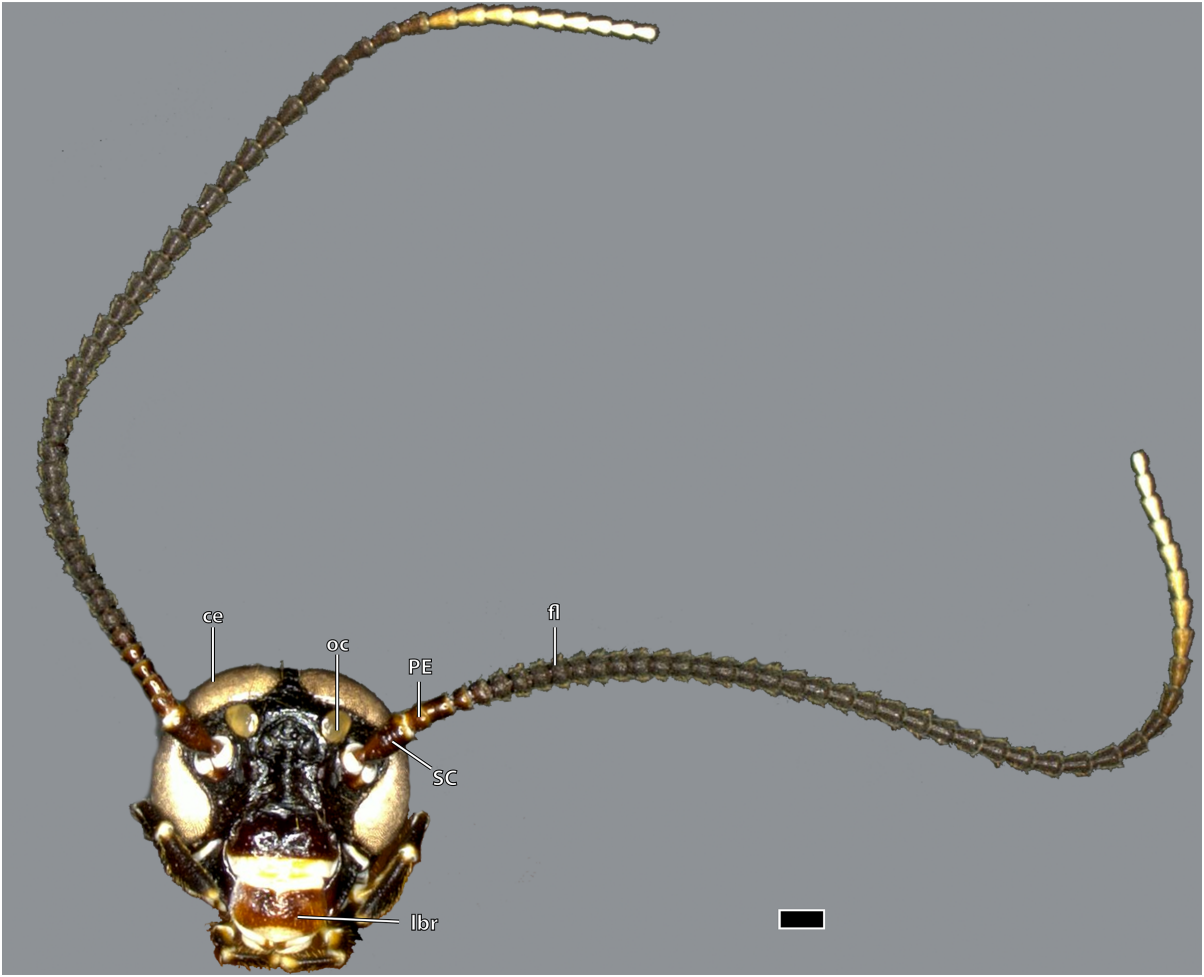
Head of *Ergaula capucina* ♂, frontal view. Scale bar: 500 µm. Abbreviations: ce, compound eye; fl, antennal flagellum; lbr, labrum; oc, ocellus; PE, pedicle; SC, scape.

**Figure 2:**
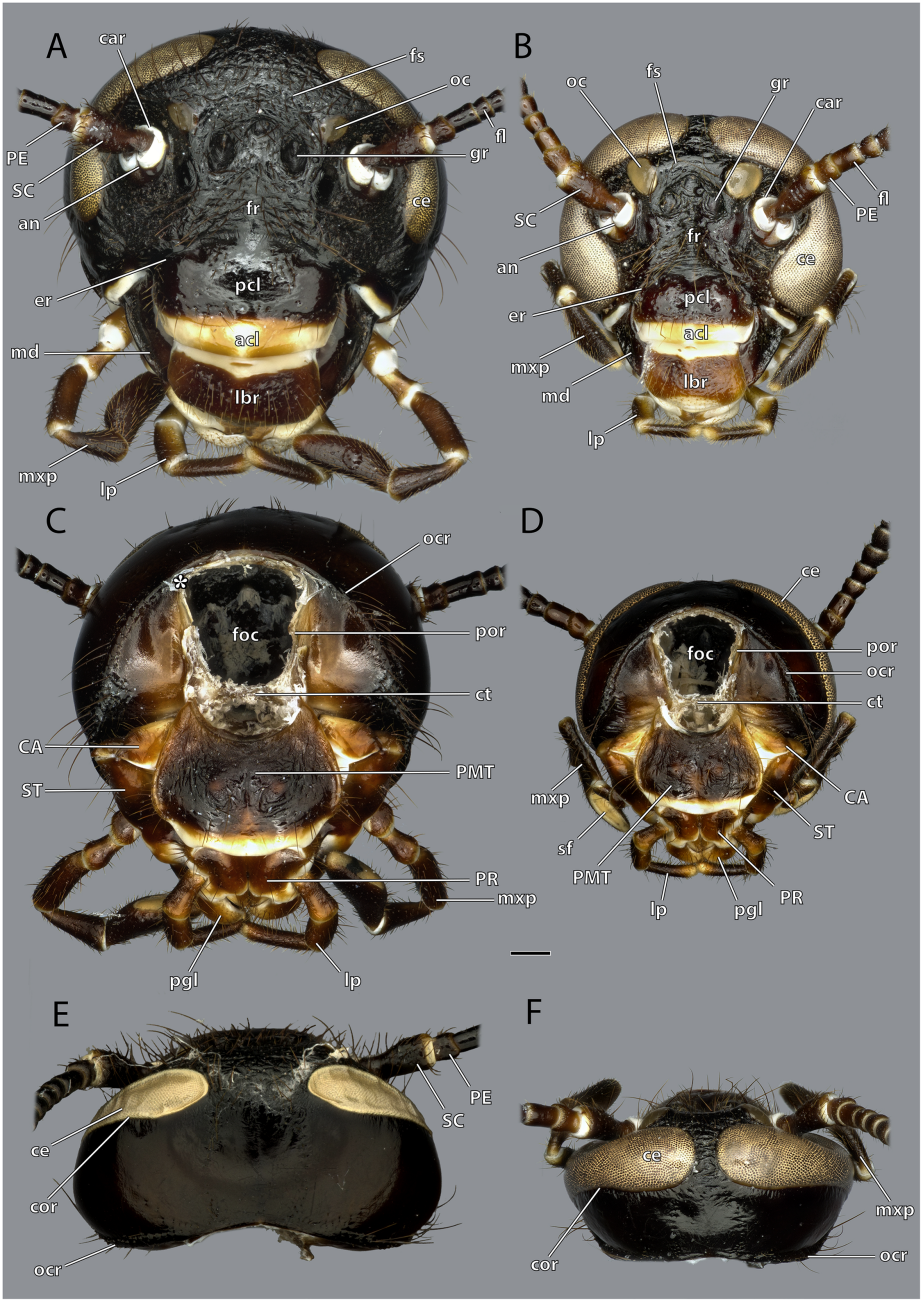
Head capsule of *Ergaula capucina*. Scale bar: 500 µm. (A) ♀ frontal view; (B) ♂ frontal view; (C) ♀ posterior view; (D) ♂ posterior view; (E) ♀ dorsal view; (F) ♂ dorsal view. Abbreviations: acl, anteclypeus; an, antennifer; CA, cardinal sclerite; car, circumantennal ridge; ce, compound eye; cor, circumocular ridge; ct, corpotentorium; er, epistomal ridge; fl, antennal flagellum; foc, foramen occipitale; fr, frons; fs, frontal cleavage line; gr, frontal pit; lbr, labrum; lp, labial palp; md, mandible; mxp, maxillary palp; oc, ocellus; ocr, occipital ridge; pcl, postclypeus; PE, pedicle; pgl, paraglossa; PMT, postmental sclerite; por, postoccipital ridge; PR, praemental sclerite; SC, scape; sf, sensory field on the maxillary palp; ST, stipital sclerite.

**Figure 3:**
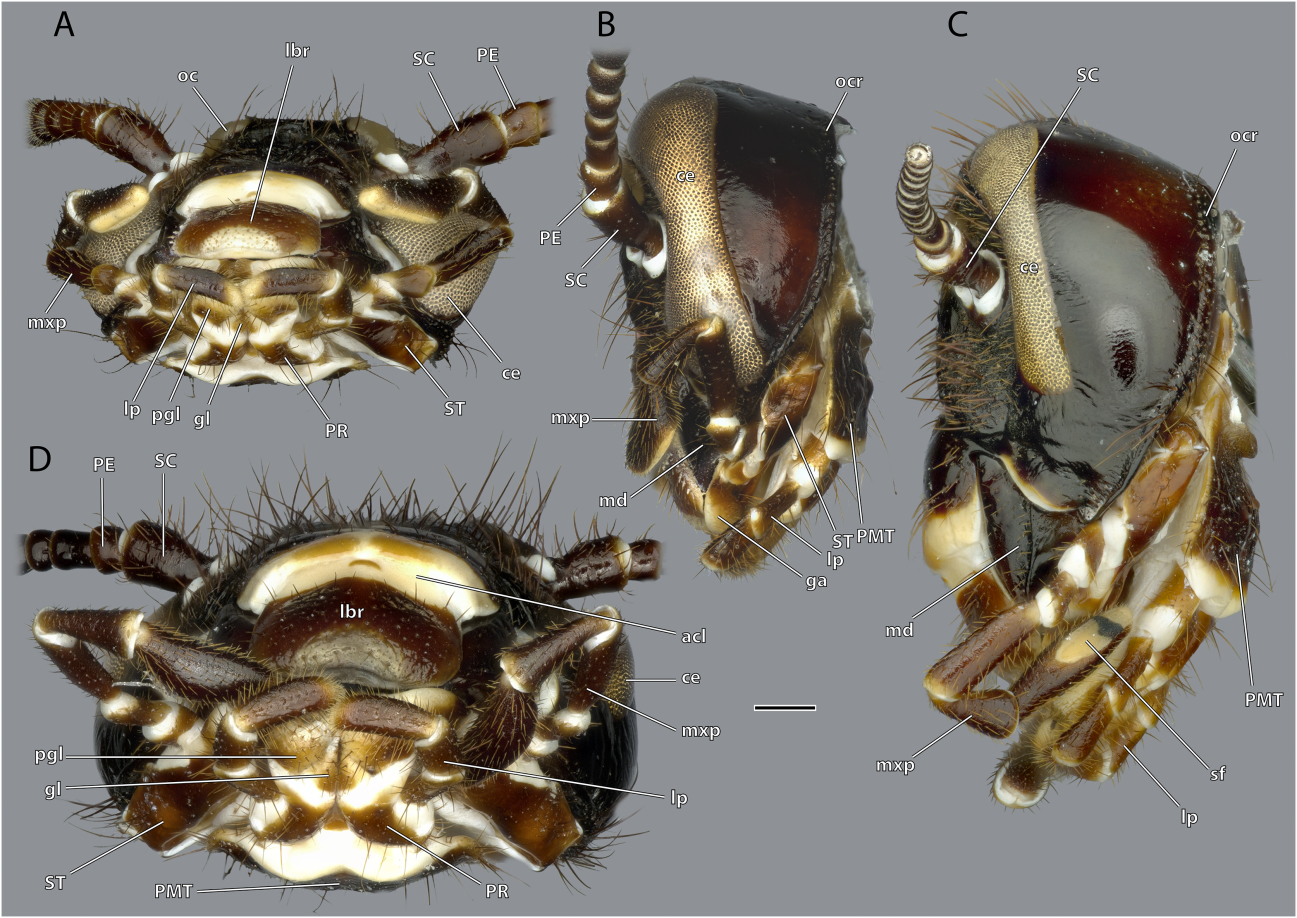
Head capsule of *Ergaula capucina*. Scale bar: 500µm. (A) ♂ ventral view; (B) ♂ lateral view; (C) ♀ lateral view; (D) ♀ ventral view. Abbreviations: ce, compound eye; gl, glossa (not labelled in male); lbr, labrum; lp, labial palp; md, mandible; mxp, maxillary palp; oc, ocellus; ocr, occipital ridge; PE, pedicle; pgl, paraglossa; PMT, postmental sclerite; PR, praemental sclerite; SC, scape; sf, sensory field on the maxillary palp; st, stipital sclerite.

**Figure 4:**
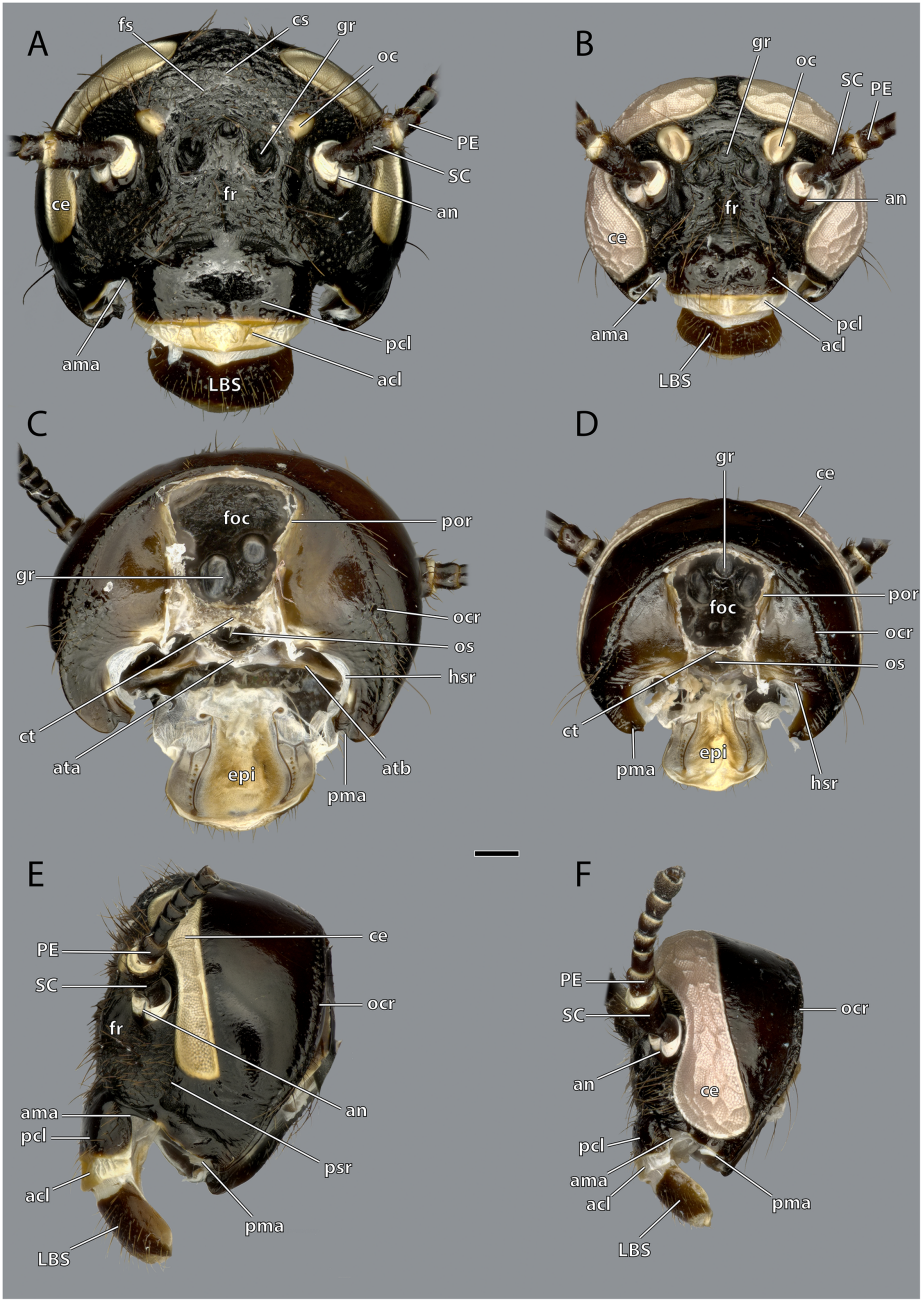
Head capsule of *Ergaula capucina* without mouthparts. Scale bar: 500µm. (A) ♀ frontal view; (B) ♂ frontal view; (C) ♀ posterior view; (D) ♂ posterior view; (E) ♀ lateral view; (F) ♂ lateral view. Abbreviations: acl, anteclypeus; ama, anterior articulation of the mandible; an, antennifer; ata, anterior tentorial arm (not labelled but present in males); atb, anterior tentorial bridge; ce, compound eye; cs, coronal cleavage line (not labelled but present in males); ct, corpotentorium; epi, epipharynx; foc, foramen occipitale; fr, frons; fs, frontal cleavage line (not labelled but present in males); gr, frontal pit; hsr, hypostomal ridge; LBS, labral sclerite; oc, ocellus; ocr, occipital ridge; os, oesotendon; pcl, postclypeus; PC, pedicle; pma, posterior articulation of the mandible; por, postoccipital ridge; psr, pleurostomal ridge; SC, scape.

**Figure 5:**
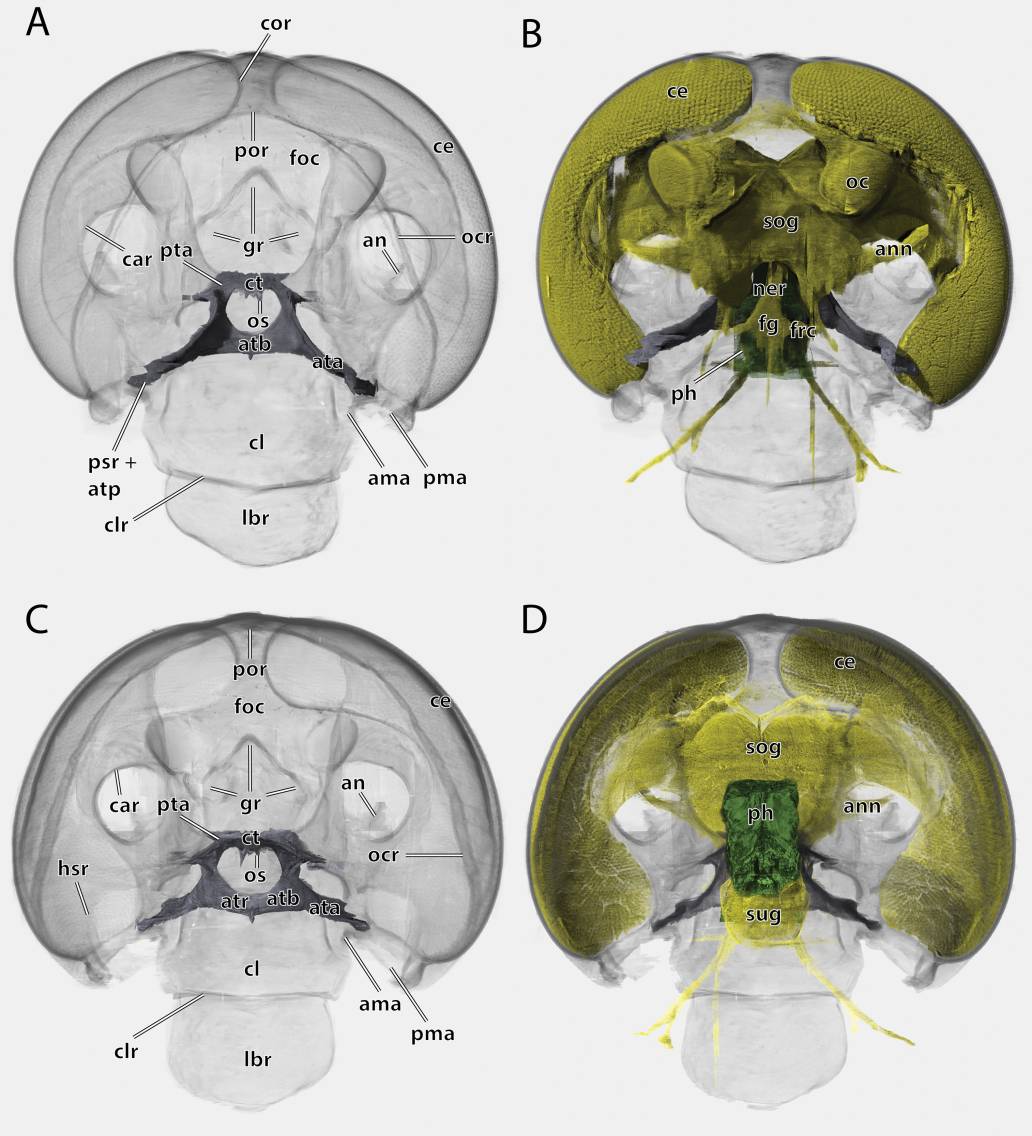
Volume render of the head of *Ergaula capucina* ♂ without mouthparts, cuticle rendered transparent. (A) Frontal view; (B) frontal view with nervous system and pharynx; (C) posterior view; (D) posterior view with nervous system and pharynx. Abbreviations: ama, anterior articulation of the mandible; an, antennifer; ann, antennal nerve; ata, anterior tentorial arm; atb, anterior tentorial bridge; atp, anterior tentorial pit; atr, anterior tentorial ridge; car, circumantennal ridge; ce, compound eye; cl, clypeus; clr, clypeo-labral ridge; cor, circumocular ridge; ct, corpotentorium; fg, frontal ganglion; foc, foramen occipitale; frc, frontal connective; gr, frontal pit; hsr, hypostomal ridge; lbr, labrum; ner, nervus recurens; oc, ocellus; ocr, occipital ridge; os, oesotendons; ph, pharynx; pma, posterior articulation of the mandible, por, postoccipital ridge; psr, pleurostomal ridge; pta, posterior tentorial arm; sog, supraoesophageal ganglion; sug, suboesophageal ganglion.

**Figure 6:**
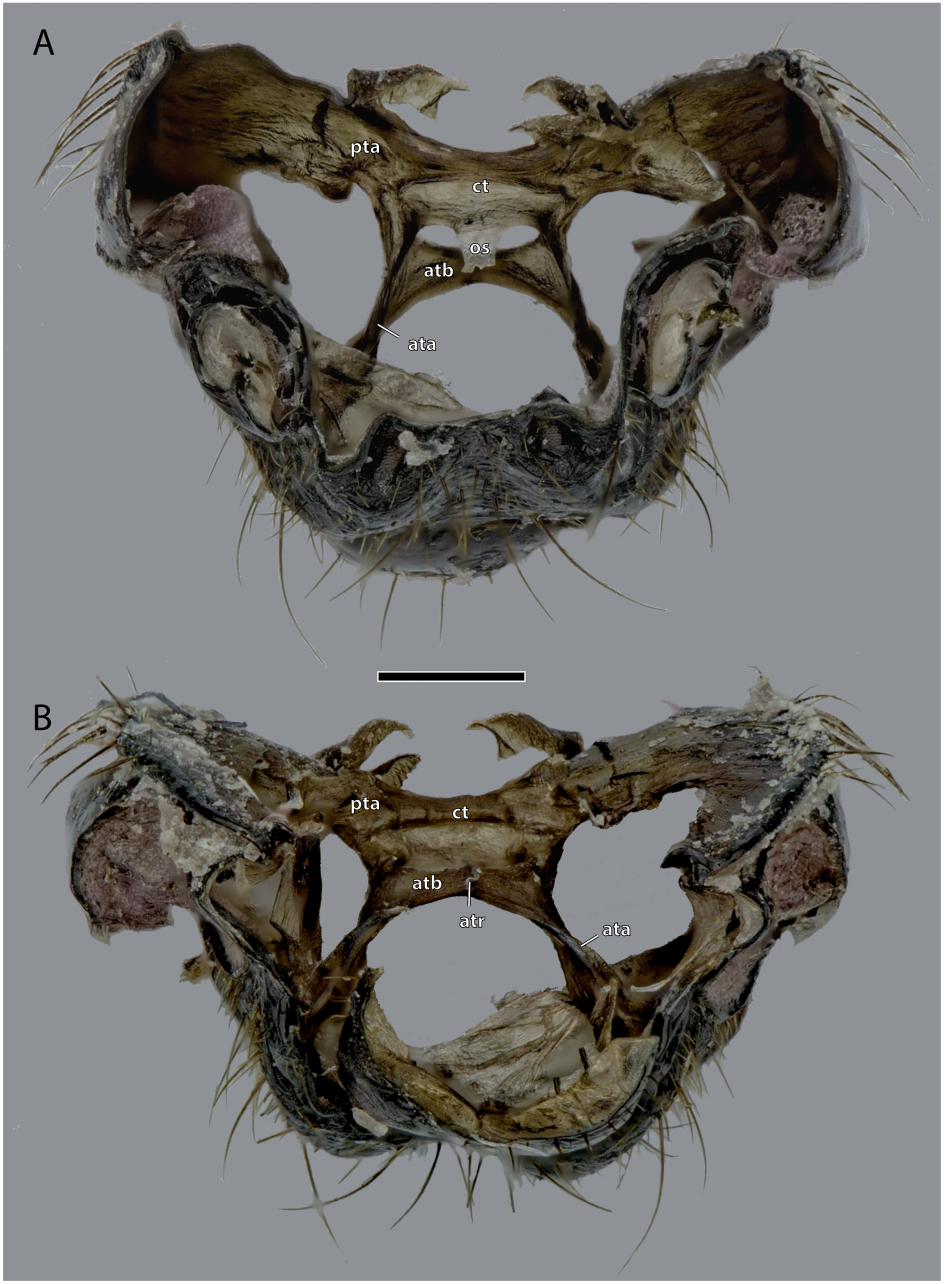
Tentorium of *Ergaula capucina* ♂; head capsule partly removed. Scale bar: 500 µm. (A) Dorsal view; (B) ventral view. Abbreviations: ata, anterior tentorial arm; atb, anterior tentorial bridge; atr, anterior tentorial ridge; ct, corpotentorium; os, oesotendons; pta, posterior tentorial arm.

**Figure 7:**
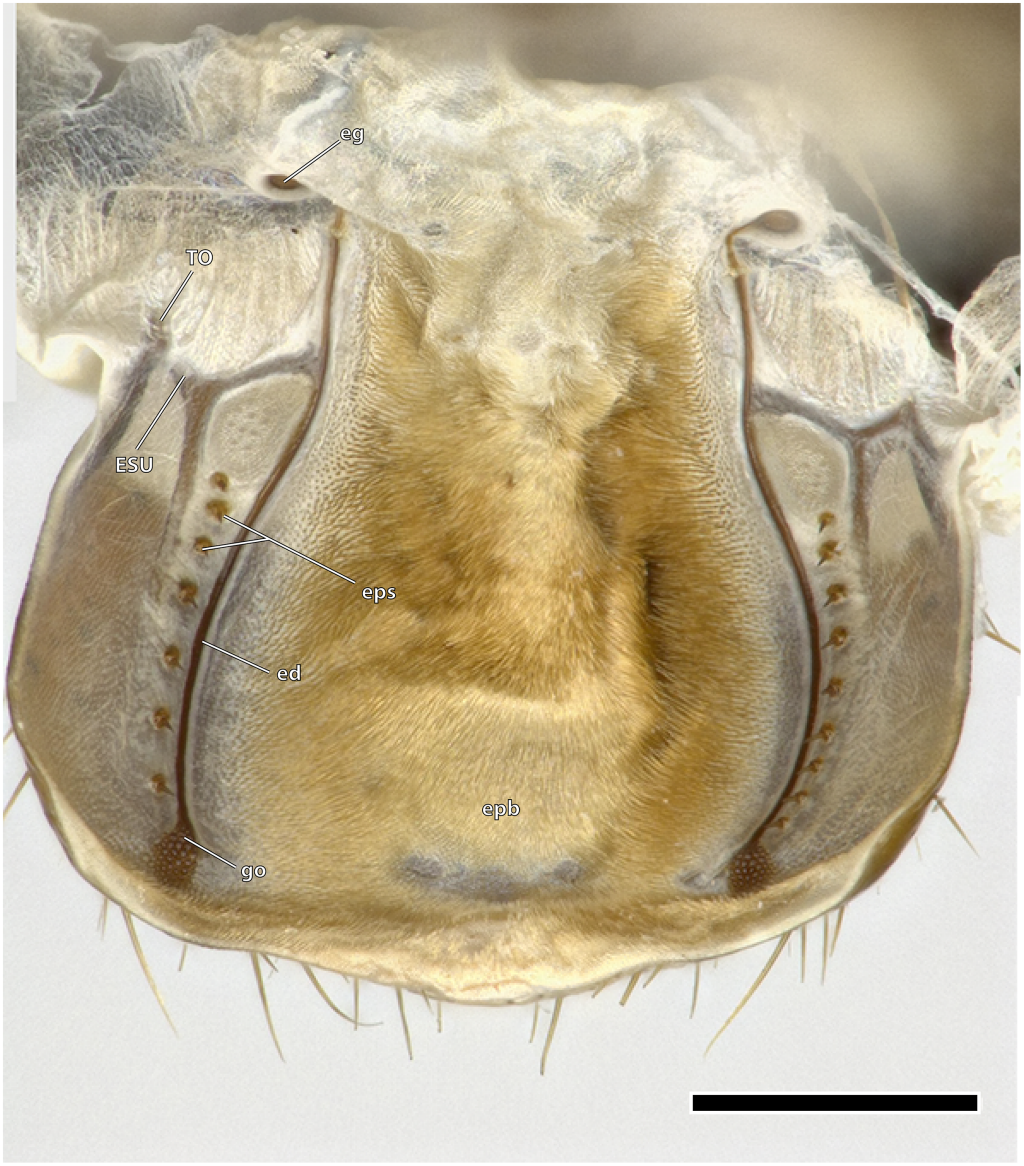
Epipharynx of *Ergaula capucina ♀*. Scale bar: 500 µm. Abbreviations: ed, ductus of the epipharyngeal gland; eg, epipharyngeal gland; epb, epipharyngeal brush; eps, epipharyngeal spines; ESU, epipharyngeal suspensorium; go, opening of the epipharyngeal gland; TO, tormae.

**Figure 8:**
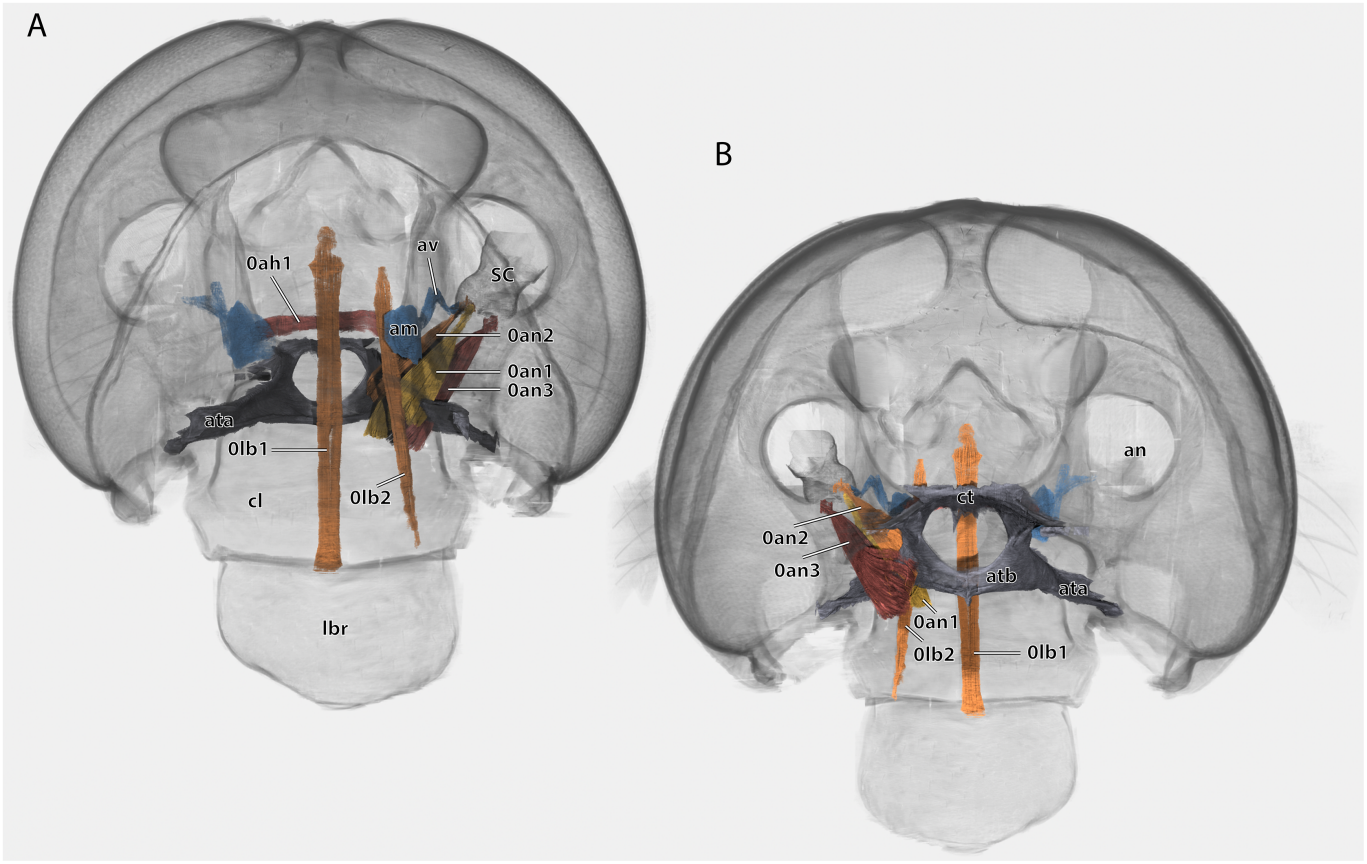
Volume render of the antennal and labral muscles of a male *Ergaula capucina*; cuticle rendered transparent and mouthparts removed. (A) Frontal view; (B) posterior view. Abbreviations: am, antennal ampulla; an, antennifer; ata, anterior tentorial arm; atb, anterior tentorial bridge; av, antennal vessel; cl, clypeus; ct, corpotentorium; lbr, labrum; SC, scape.

**Figure 9:**
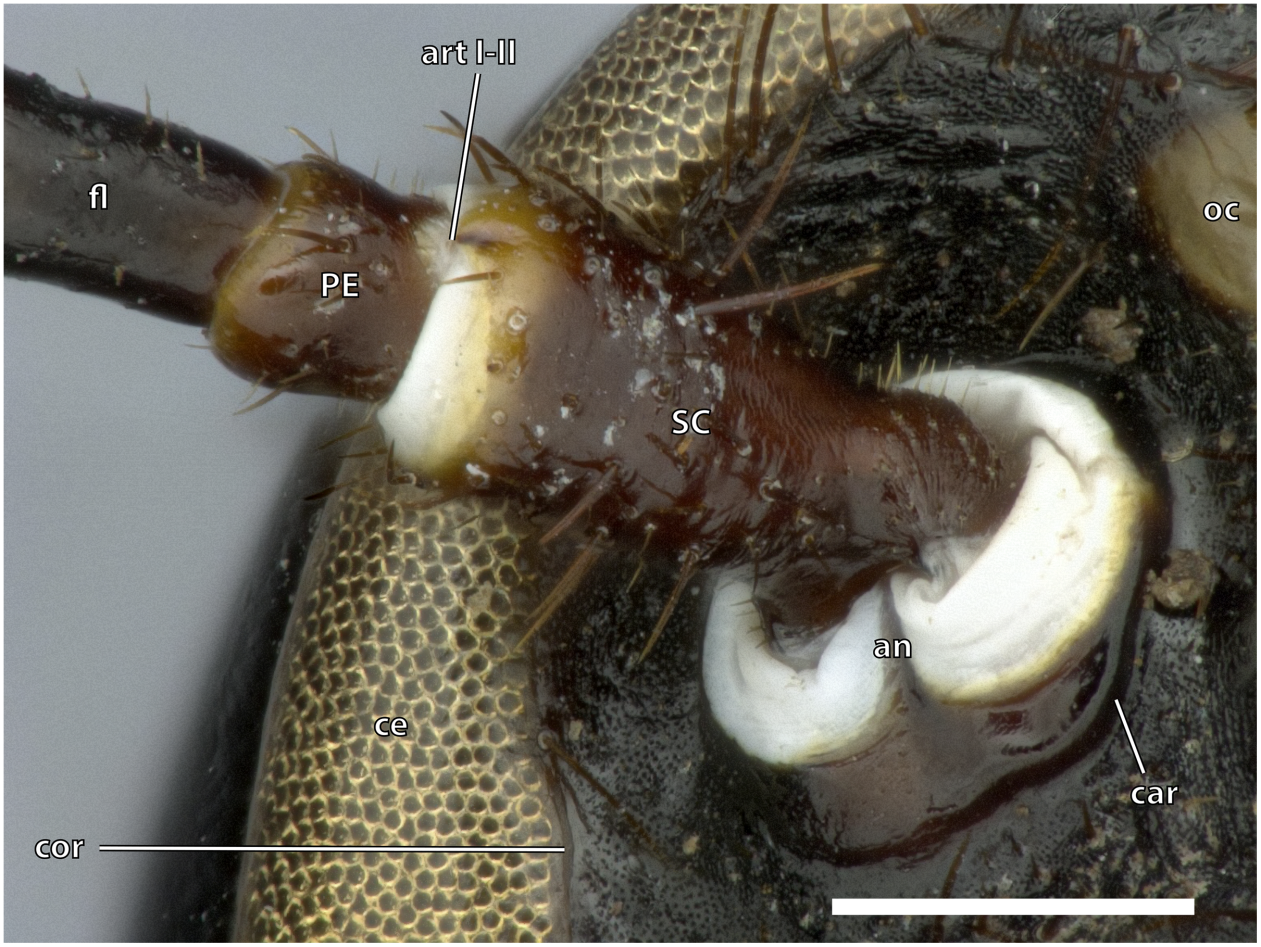
Anntenal base of *Ergaula capucina ♀*. Scale bar: 500 µm. Abbreviations: an, antennifer; art I-II, median articulation between scape and pedicle; car, circumantennal ridge; ce, compound eye; cor, circumocular ridge; fl, antennal flagellum, oc, ocellus; PE, pedicle, SC, scape.

The orthognathous head capsule (Figs. 1–7) of the female is flattened in antero-posterior direction and reversely drop-shaped with the maximum width on the level of the antennal bases. Its coloration is reddish-brown to blackish-brown. The head capsule is almost entirely covered with distinct setae that are shorter than the scape (SC, Figs. 1–4, 8, 9). On the dorsal area of the posterior surface around the occipital ridge, the setae are distinctly longer (approximately as long as the scape).

The large and nearly squared foramen occipitale (foc, Figs. 2, 4, 5) is located on the posterior side of the head capsule and forms the connection between the head capsule and the lumen of the thorax. It is surrounded by the postoccipital ridge (por, Figs. 2, 4, 5). Dorso-lateral on this ridge, a postoccipital condyle is positioned on each side. The occipital ridge (ocr, Figs. 2–5) runs distinctly separated from the foramen- over the posterior head capsule and ends ventrally on both sides near the posterior articulation of the mandible (pma, Figs. 4, 5).

The kidney-shaped compound eyes (ce, Figs. 1–5, 9) of the female are positioned laterally on the dorsal part of the head capsule. Each eye is surrounded by a circumocular ridge (cor, Figs. 2, 5, 9). The eyes partially surround the antennal sockets. Each socket is surrounded by a circumantennal ridge (car, Figs. 2, 5, 9), which is distinctly separated from the circumocular one. The articulation of the antenna with the head capsule is achieved *via* an antennifer (an, Figs. 2, 4, 5, 8, 9) that is embedded in the ventral margin of the antennal socket. The distance between the two antennal sockets is approximately 3 times as wide as the diameter of one socket. Two lateral ocelli (oc, Figs. 1–5, 9) are located mesally of the upper half of the compound eyes and meso-dorsally of the antennal base. The median ocellus is missing. On the ventral side of the compound eyes the pleurostomal ridge (psr, Figs. 2, 4, 5) separates from the circumocular ridge. Its posterior part runs ventro-posteriorly towards the posterior mandibular articulation while the anterior part runs ventro-mesally where it unites with anterior mandibular articulations that form exteriorly visible pits (atp, Fig. 5). The arch-shaped pleurostomal ridge thus divides the space below the eye in the dorsal gena and the ventral subgena. From the anterior mandibular articulation a short and weaker epistomal ridge continues. The epistomal ridges of both sides do not unite in the middle. From the posterior mandibular articulations (pma, Figs. 4, 5), the hypostomal ridge (hsr, Figs. 4, 5) continues and forms the articulations with the maxillae and the labium. It ends at the posterior tentorial pits from where it continues as postoccipital ridge around the foramen occipitale.

Due to the mesally separated epistomal ridge, the frons (fr, Figs. 2, 4) is not clearly delimited from the ventral clypeus (cl, Figs. 5, 8). The frons is densely covered with long setae. The clypeus is trapezoid and bears several setae which are shorter than those on the frons. It is divided in a strongly sclerotized postclypeus (pcl, Figs. 2, 4) and a weaker sclerotized anteclypeus (acl, Figs. 2, 4). The ventral margin of the clypeus is reinforced by the clypeo-labral ridge (clr, Fig. 5).

The coronal cleavage line runs mid-sagitally from the postoccipital ridge over the head capsule. In between the compound eyes it separates into two frontal cleavage lines (fs, Figs. 2, 4) which end near the ocelli. Medio-ventrally between the ocelli three frontal pits (gr, Figs. 2, 4, 5) are located. The upper one, which forms the attachment of M. frontolabralis (0lb1) is mid-sagittal while the two lower ones are positioned laterally below the former and serve as attachment for M. frontoepipharyngealis (0lb2).

The head capsule of the male (Figs. 1–3) is distinctly smaller than the female one. Further difference is the much bigger compound eyes and ocelli. The male’s eyes reach far more ventrally than the female’s eyes and are almost in contact with the anterior mandibular articulations. The setae on the male frons are relatively less dense and shorter than their female counterparts. In all other described respects, they are similar to the female.

### Tentorium

The tentorium is composed of paired anterior (ata, Figs. 4–6, 8) and posterior tentorial arms (pta, Figs. 5, 6) which are connected *via* the unpaired corpotentorium (ct, Figs. 2, 4–6, 8) and the anterior tentorial bridge (atb, Figs. 4–6, 8). Dorsal tentorial arms are not present. The large anterior tentorial arms originate from the anterior tentorial pits and are twisted approximately 120° (the left one clockwise, the right one counter-clockwise). Their margins on both sides are reinforced with bulges. In the middle of their lengths, both anterior arms fuse to the anterior tentorial bridge, which has a median crest (atr, Figs. 5, 6) on its ventral side. In the posterior third, they separate again, thus forming the “perforation” of the tentorium. Posteriorly the corpotentorium attaches. It is dorso-ventrally flattened and on a slightly more dorsal plane than the anterior tentorial bridge. On its anterior margin, the paired laminar oesotendons (os, Figs. 4–6) serve as attachment for M. tentoriosuspensoralis (0hy5) and protrude into the “perforation”. On the postero-lateral corners of the corpotentorium, the posterior tentorial arms attach and run towards to the posterior tentorial pits. The tentorium serves as muscle attachment site for various muscles listed below.

### Labrum and epipharynx

The labrum (lbr, Figs. 1–3, 5, 8) is trapezoid and is located distally to the clypeus. The anterior surface of the labrum is formed by the labral sclerite (LBS, Fig. 4) which is covered with long setae.

The epipharynx (epi, Figs. 4, 7) forms the inner, posterior wall of the labrum and clypeus and frontal wall of the mouth cavity. Dorsally it ends at the functional mouth opening. The epipharynx is generally membranous although several sclerotized structures are embedded in it. The tormae are sclerotized bars positioned laterally at the labral base. They are firmly connected with the labral sclerite and serve as attachment site for M. frontoepipharyngealis. Each torma is connected with an epipharyngeal suspensorium (ESU, Fig. 7), that has a distally and a mesally protruding arm. Each mesal arm is connected to the sclerotized ductus of the epipharyngeal gland (ed, Fig. 7) (“labral” gland of Zhuzhikov, 2007). These epipharyngeal glands (eg, Fig. 7) are located in membranous folds on each side of the animal on the clypeal part of the epipharynx. From there, sclerotized ducts run over the epipharynx towards the gland openings (go, Fig. 7) in the distal third of the labrum. A row of seven to nine epipharyngeal spines (eps, Fig. 7) is positioned laterally to the duct on each side of the animal. The median area of the epipharynx in-between the glandular ducts is covered by an epipharyngeal brush (epb, Fig. 7) made of microtrichia.

**Figure 10:**
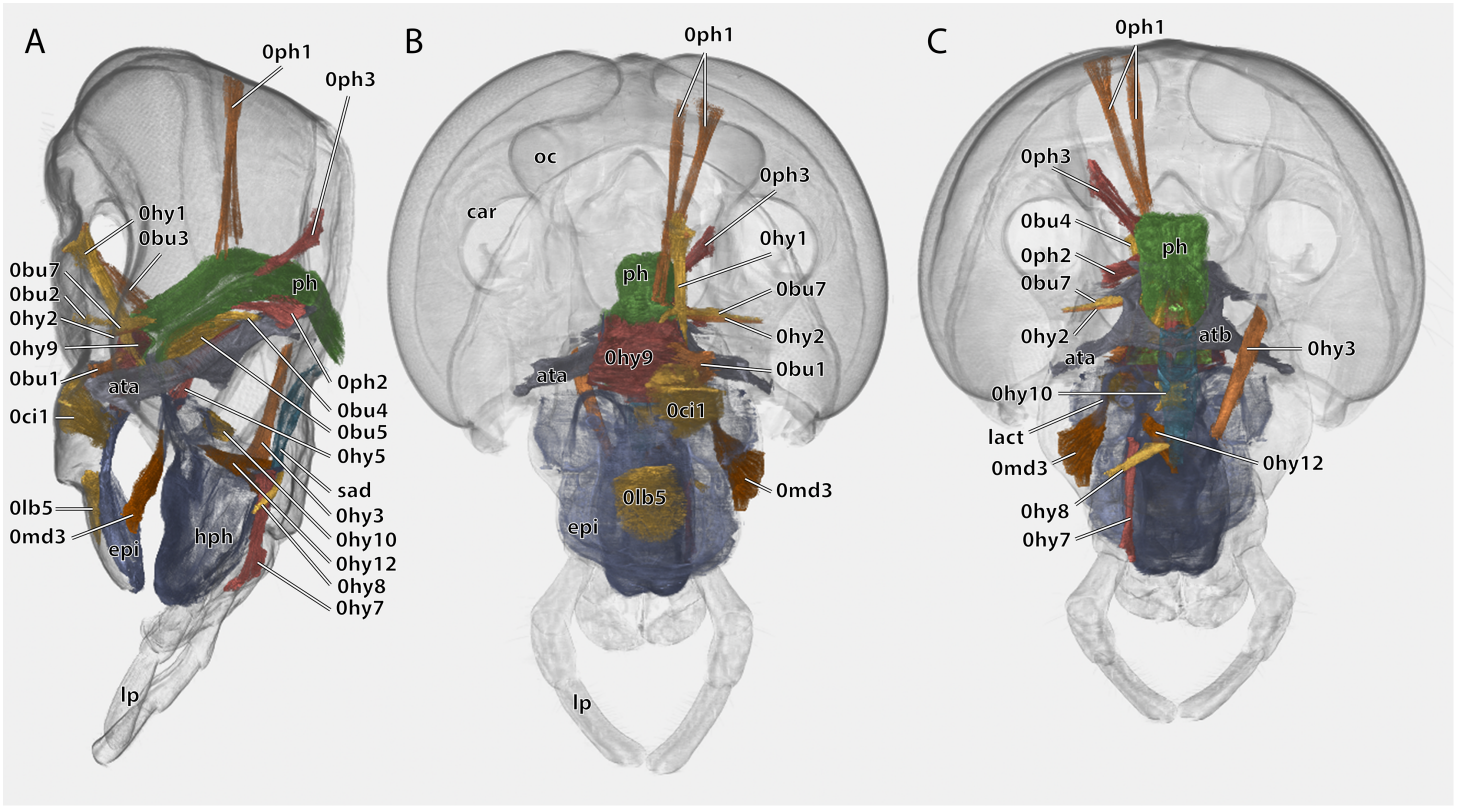
Volume render of the cephalic musculature of *Ergaula capucina*. Cuticle rendered transparent, all mouthparts except hypopharynx and labium removed. (A) Lateral view, (B) frontal view; (C) posterior view. Abbreviations: ata, anterior tentorial arm; atb, anterior tentorial bridge; car, circumantennal ridge; epi, epipharynx; hph, hypopharynx; lact, linguactual apodem; lp, labial palp; oc, ocellus; ph, pharynx; sad, salivary ductus.

Musculature (Figs. 8 and 10): M. frontolabralis (0lb1)–Origin (O): mesally on the frons; Insertion (I): mesally on the frontal wall of the labrum. M. frontoepipharyngalis (0lb2)–O: mesally on the frons, slightly laterally of M. frontolabralis; I: on the tormae. M. labralis transversalis (0lb4, not figured)–O: ventral labral wall; I: ventral labral wall of the opposite side. M. labroepipharyngealis (0lb5)–O: inner labral wall, ventrally of the insertion of M. frontolabralis; I: inner epipharyngeal wall, laterally and slightly ventrally of the origin. M. clypeopalatalis (0ci1), strongly developed–O: mesally on the clypeus; I: on the epipharyngeal gland on the epipharynx.

### Antennae

The antennae are 3 to 4 times as long as the head capsule and articulate with it *via* antennifers. The scape (SC, Figs. 1–4, 9) is a cylinder-shaped sclerite approximately 3 times as long as wide and sparsely covered with setae. The pedicle (PE, Figs. 1–4, 9) is around a third as long as the scape. The two segments are connected *via* distinct median articulations (art I-II, Fig. 9). At its base, the antennal flagellum (fl, Figs. 1, 2, 9) has approximately the same diameter as the pedicle and comprises approximately 40 flagellomeres, which are sparsely covered with setae. Flagellomere 1 is twice as long as the pedicle while all following ones are approximately the same size. The distal 10 segments narrow increasingly and transit to a more moniliform shape in contrast to the more filiform base. The paired antennal ampullae (am, Fig. 8) are attached to the frons mesally of the antennal base at the approximate level of the antennifers. The antennal vessels run from the ampullae into the antennae.

Musculature (Fig. 8): M. tentorioscapalis anterior (0an1)–O: meso-frontally on the anterior tentorial bridge; I: ventro-mesally on the scapal base. M. tentorioscapalis posterior (0an2)–O: laterally on the corpotentorium and the anterior tentorial bridge; I: dorsally at the scapal base. M. tentorioscapalis lateralis (0an3)–O: anterior tentorial arm; I: ventro-laterally at the scapal base. M. interampullaris (0ah1)–O: mesal side of antennal ampulla; I: mesal side of antennal ampulla on the opposite side. M. ampulloaortica (0ah2, not figured)–O: mesal side of antennal ampulla, together with M. interampullaris; I: anterior end of the aorta.

### Mandibles

**Figure 11:**
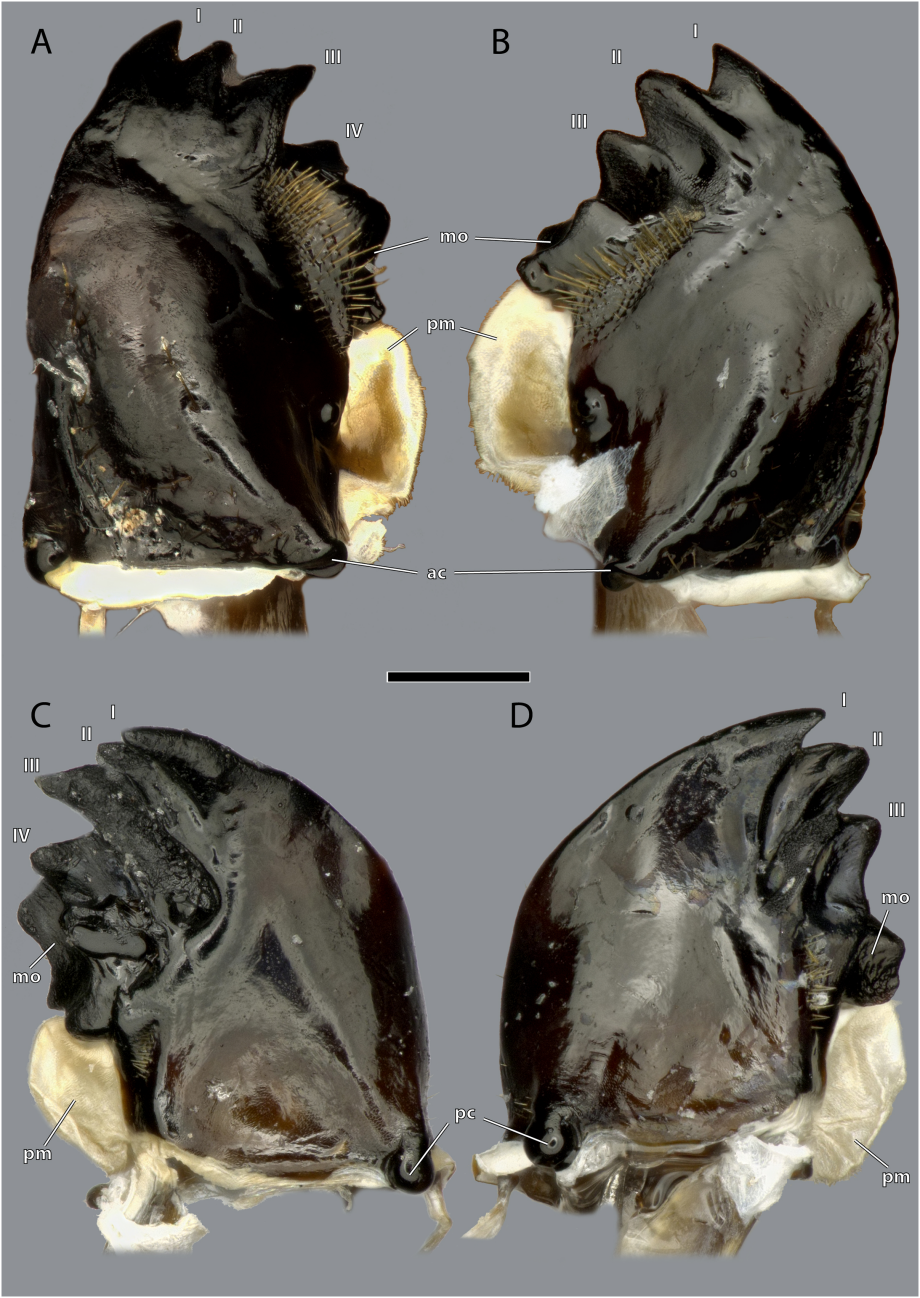
Mandibles of *Ergaula capucina* ♀. Scale bar: 500 µm. (A) Left mandible, frontal view; (B) right mandible, frontal view; (C) left mandible, posterior view; (D) left mandible, posterior view. Abbreviations: I–IV, mandibular incisivi; ac, anterior mandibular condyle; mo, molar region of the mandible; pc, posterior mandibular condyle; pm, postmola.

**Figure 12:**
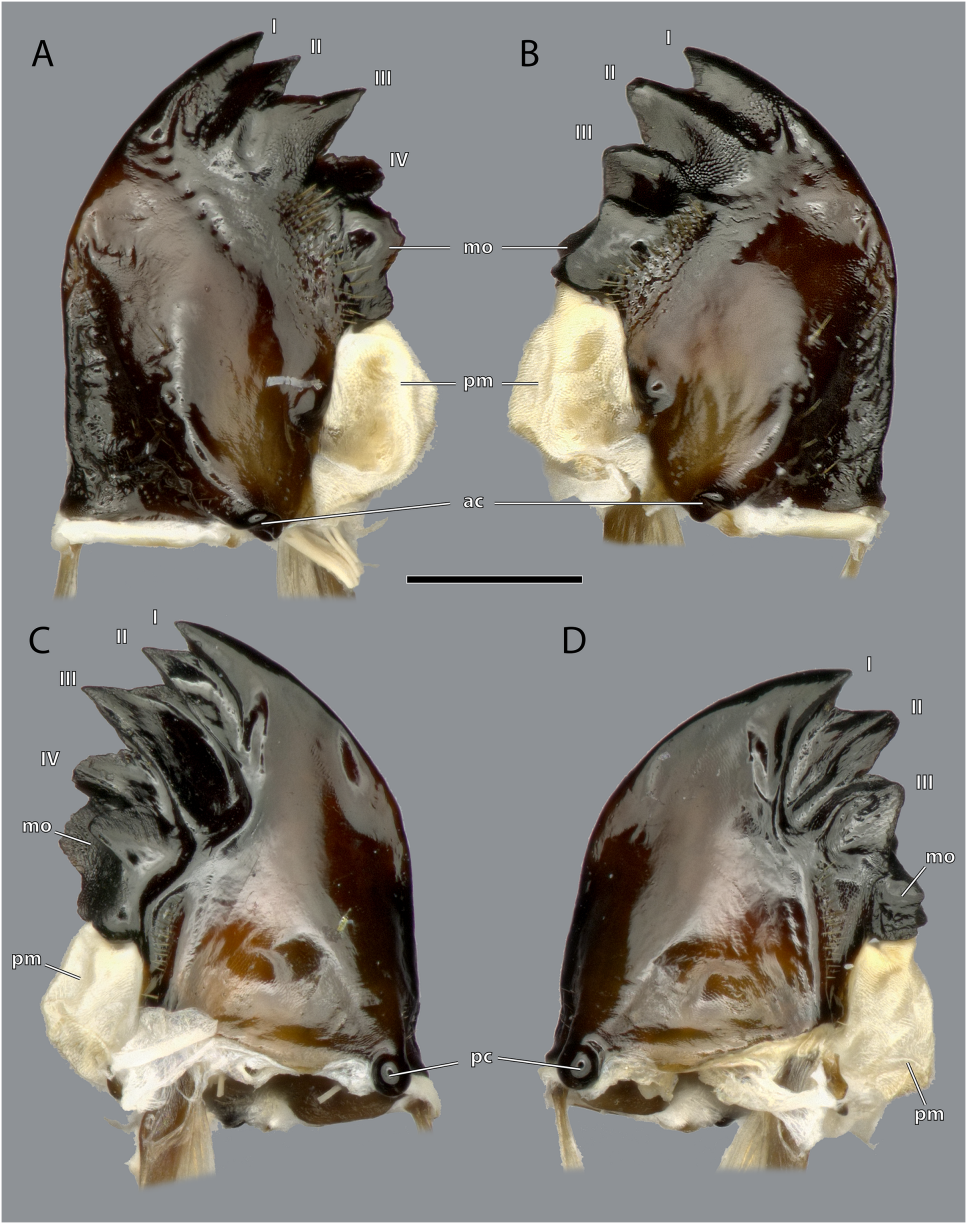
Mandibles of *Ergaula capucina* ♂. Scale bar: 500 µm. (A) Left mandible, frontal view; (B) right mandible, frontal view; (C) left mandible, posterior view; (D) right mandible, posterior view. Abbreviations: I–IV, mandibular incisivi; ac, anterior mandibular condyle; mo, molar region; pc, posterior mandibular condyle; pm, postmola.

**Figure 13:**
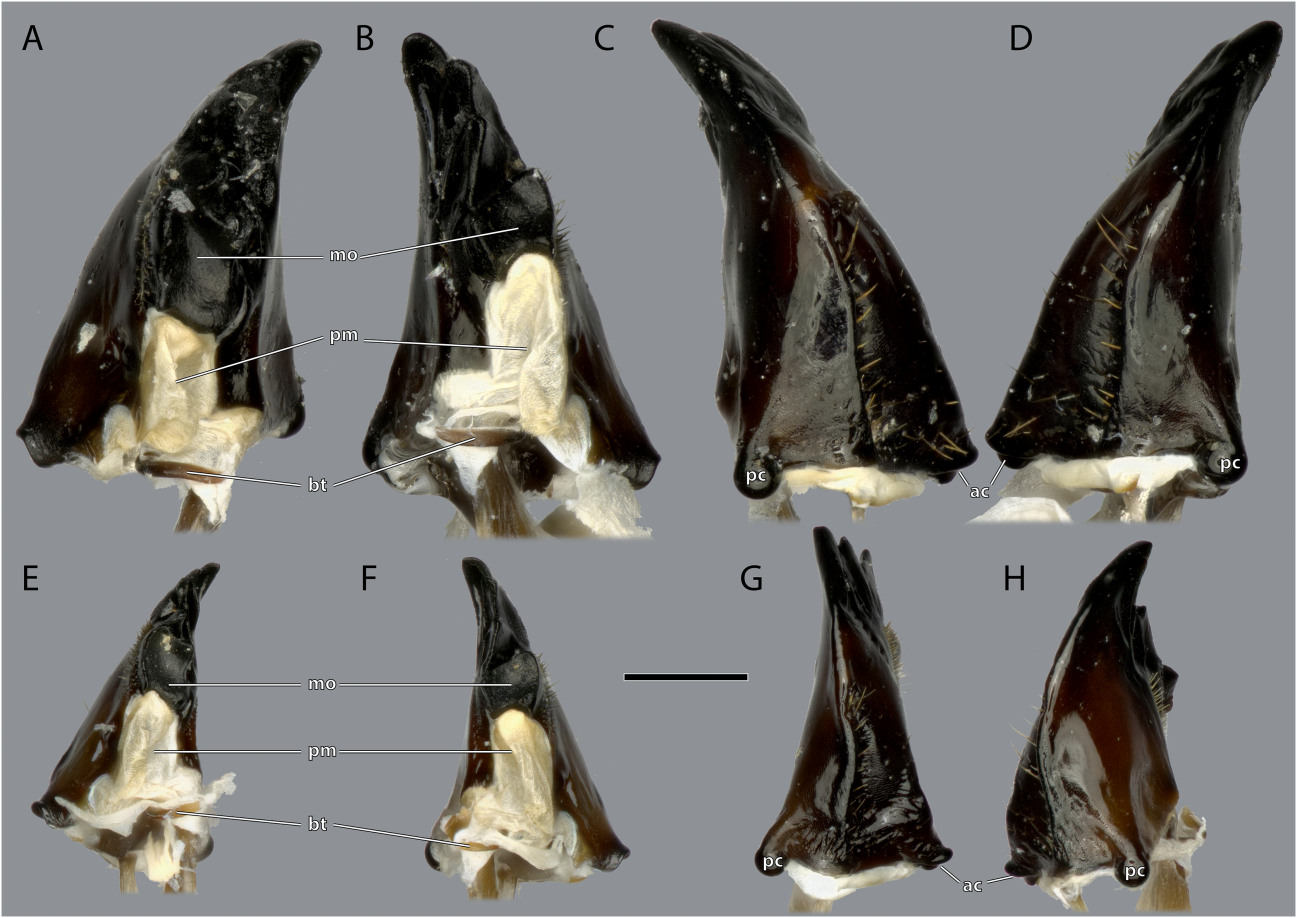
Mandibles of *Ergaula capucina*. (A) ♀ Left mandible, mesal view; (B) ♀ right mandible, mesal view; (C) ♀ left mandible, lateral view; (D) ♀ right mandible, lateral view; (E) ♂ left mandible, mesal view; (F) ♂ right mandible, mesal view; (G) ♂ left mandible, lateral view; (H) ♂ right mandible, lateral view. Scale bar: 500 µm. Abbreviations: ac, anterior mandibular condyle; bt, basatendon; mo, molar region; pc, posterior mandibular condyle; pm, postmola.

**Figure 14:**
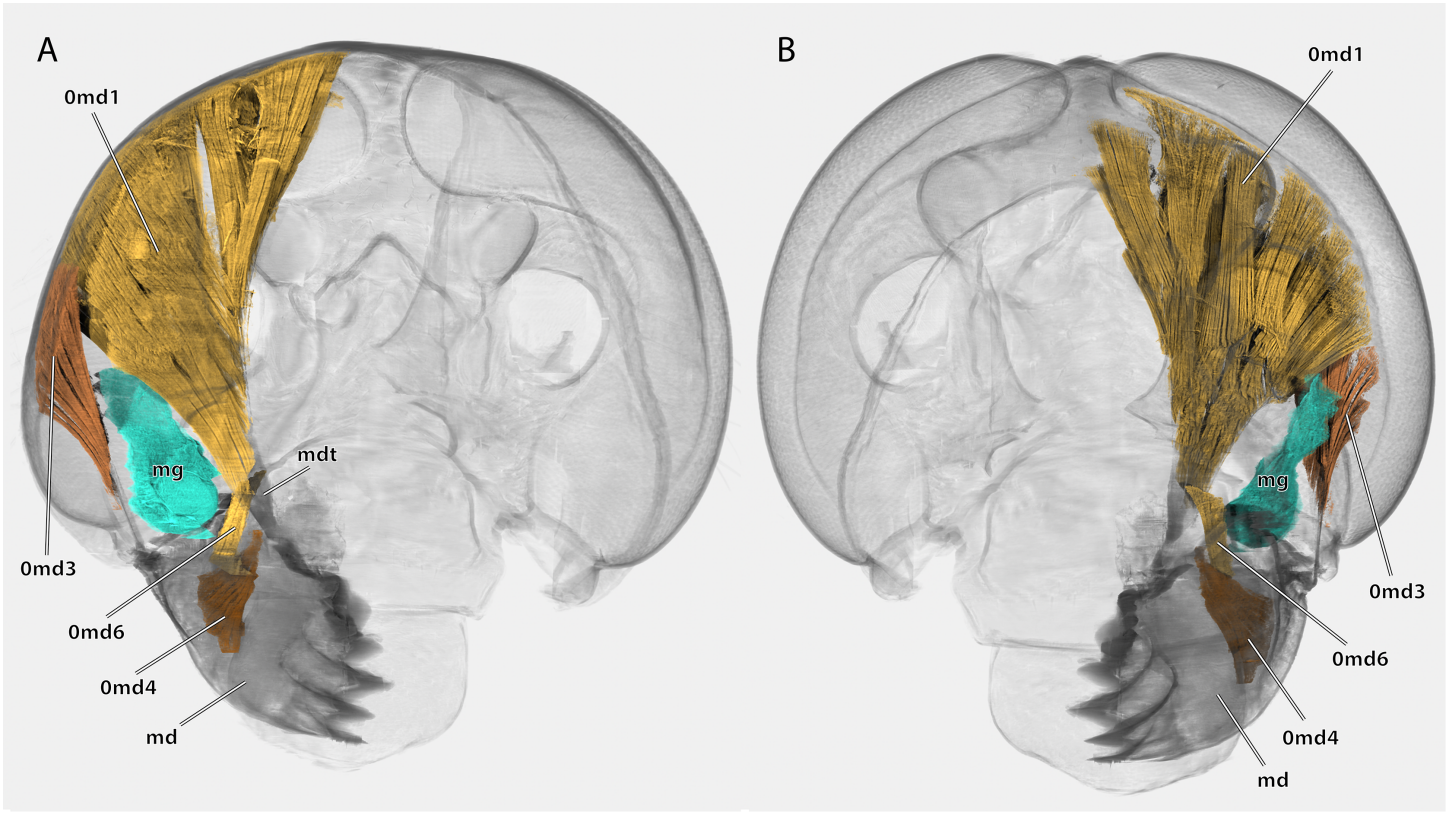
Volume render of the mandibular musculature of *Ergaula capucina* ♂. Cuticle rendered transparent, mouthparts except mandibles removed. (A) posterior view; (B) anterior view. Abbreviations: md, mandible; mdt, mandibular tendon; mg, mandibular gland.

Each of the two asymmetric mandibles (md, Figs. 2, 3, 11–14) is composed of a single sclerite that has a triangular shape when seen frontally. In their distal third they show a posterior bend. The mandibles articulate *via* two ball-and-socket-joints with the head capsule. The mandible forms the socket for the anterior (ama, Figs. 4, 5) and the ball for the posterior joint (pma, Figs. 4, 5).

The left mandible has four distal incisivi (I–IV, Fig. 11), from which the third one (III) is the biggest and the fourth one (IV) the smallest. Table 1 provides an overview over the mechanical advantage of all incisivi. Proximally to the incisivi the mola is located on the mesal side. In the left mandible the mola forms a concave area approximately twice as high as wide. Proximally of the mola, the membranous and sail-shaped postmola attaches. It is densely covered with microtrichia. On the posterior side of the mandible a sclerotized plate (the basatendon of Zhuzhikov, 2007) is embedded into the membrane near the articulation with the tendon of M. craniomandibularis internus (0md1). This tendon of the mandibular adductor starts at the postero-mesal base of the mandible and reaches far into the lumen of the head capsule where it fans into two major branches: a mesally orientated and rather squared one and a larger laterally orientated flattened branch. On the frontal surface of the mandible, an area right next to the mandibular mola and incisivus IV is densely covered with setae. A similar but smaller area is also present on the posterior side.

**Table 1:** Mechanical advantage (MA) of the mandible of *Ergaula capucina*.

		MA
**Right**	Incisivus 1	0.37
	Incisivus 2	0.39
	Incisivus 3	0.45
**Left**	Incisivus 1	0.37
	Incisivus 2	0.40
	Incisivus 3	0.41
	Incisivus 4	0.46
	Incisivus 5	0.52

The right mandible has only three incisivi of which the proximal one is also the smallest and the second one is both the strongest and longest. The mola of the right mandible is convex, thus perfectly fitting in the concave left one.

Musculature (Fig. 14): M. craniomandibularis internus (0md1), the strongest muscle in the head capsule–O: in eight different bundles (described in Table 2) along the dorsal and dorso-lateral head capsule; I: with a massive tendon on the mesal edge of the mandibular base. Table 3 provides the respective effective cross section areas and volumes of these bundles for both body sides. M. craniomandibularis externus posterior (0md3)–O: lateral head capsule, posteriorly of the compound eye; I: with a long tendon on the lateral basal mandibular edge. Table 3 provides the respective effective cross-section areas and volumes of the muscle for both body sides. M. hypopharyngomandibularis (0md4)–O: distal tip of the linguactual apodeme of the hypopharynx; I: laterally on the anterior inner mandibular wall. M. tentoriomandibularis lateralis inferior (0md6)–O: posterior side of the anterior tentorial arm, directly before the anterior tentorial bridge; I: meso-proximally on the posterior inner mandibular wall.

**Table 2:** Bundles of M. craniomandibularis internus in *Ergaula capucina*.

Muscle bundle	Origin	Insertion
a	Laterally on basal and mesal wing of the adductor tendon	Latero-posterior vertex, directly posterior of the compound eye
b	Posterior side of the basal wing & ventral parts of lateral and mesal wing	Laterally of the foramen occipitale on the postgena
c	Mesal side of the lateral wing	Vertex, mesal to a and laterally of d and e
d	In three bundles along the mesal wing	Anterior vertex
e	Laterally on the mesal wing	Posterior vertex in the left hemisphere; posterior of g
f	Mesal and lateral side of the mesal wing	Dorsal Vertex
g	Mesal side of the the mesal wing in between f and h	Posterior vertex
h	Mesal side of the mesal wing	Dorsal edge of the foramen occipitale

**Table 3:** Effective cross section and volume of the bundles of M. craniomandibularis internus (0md1) and M. craniomandibularis externus posterior (0md3) in different cockroaches.

	Muscle	Bundle	Area (mm^2^)		Area (%)		Volume (mm^3^)		Volume (%)			Muscle	Bundle	Area (mm^2^)		Area (%)		Volume (mm^3^)		Volume (%)	
			Mean	Std (±)	Mean	Std (±)	Mean	Std (±)	Mean	Std (±)				Mean	Std (±)	Mean	Std (±)	Mean	Std (±)	Mean	Std (±)
***Ergaula capucina* male**	**0md1 left**	Complete	0.752	–	100.000	–	0.278	–	100.000	–	***Periplaneta americana***	**0md1 left**	Complete	2.098	0.247	100.000	0.000	2.779	0.540	100.000	0.000
		a_l	0.234	–	31.156	–	0.093	–	33.319	–			a_l	0.532	0.051	25.442	1.252	0.726	0.091	26.428	2.524
		b_l	0.140	–	18.583	–	0.057	–	20.452	–			b_l	0.492	0.111	23.248	2.845	0.561	0.147	20.018	1.727
		c_l	0.093	–	12.326	–	0.037	–	13.184	–			c_l	0.320	0.081	15.117	2.409	0.556	0.221	19.398	4.668
		d_l	0.075	–	9.984	–	0.013	–	4.647	–			d_l	0.154	0.022	7.339	0.273	0.135	0.022	4.898	0.524
		e_l	0.141	–	18.738	–	0.045	–	16.059	–			e_l	0.262	0.072	12.892	5.288	0.394	0.011	14.656	3.610
		f_l	0.025	–	3.380	–	0.012	–	4.449	–			f_l	0.158	0.056	7.433	1.995	0.174	0.040	6.250	0.505
		g_l	0.035	–	4.675	–	0.015	–	5.207	–			g_l	0.154	0.018	7.339	0.196	0.181	0.035	6.530	0.310
		h_l	0.009	–	1.158	–	0.007	–	2.683	–			h_l	0.025	0.008	1.190	0.281	0.051	0.024	1.822	0.695
	**0md1 right**	Complete	1.172	–	100.000	–	0.469	–	100.000	–		**0md1 right**	Complete	2.202	0.354	100.000	0.000	3.583	0.328	100.000	0.000
		a_r	0.277	–	23.619	–	0.154	–	32.757	–			a_r	0.532	0.079	22.558	2.951	0.828	0.067	23.130	1.201
		b_r	0.224	–	19.120	–	0.077	–	16.460	–			b_r	0.508	0.077	21.484	1.554	0.671	0.044	18.769	1.097
		c_r	0.111	–	9.490	–	0.044	–	9.367	–			c_r	0.209	0.183	14.301	2.857	0.639	0.074	17.838	1.262
		d_r	0.128	–	10.944	–	0.037	–	7.807	–			d_r	0.260	0.034	11.025	0.883	0.320	0.021	8.957	0.843
		e_r	0.348	–	29.685	–	0.127	–	27.072	–			e_r	0.503	0.088	21.271	2.522	0.811	0.143	22.531	2.120
		f_r	0.047	–	4.003	–	0.014	–	2.945	–			f_r	0.067	0.058	4.093	0.349	0.138	0.037	3.805	0.689
		g_r	0.016	–	1.330	–	0.006	–	1.319	–			g_r	0.080	0.027	3.420	1.306	0.108	0.030	3.051	0.993
		h_r	0.021	–	1.810	–	0.011	–	2.273	–			h_r	0.044	0.016	1.849	0.670	0.069	0.021	1.921	0.524
	**0md3 l**	–	0.053	–	100.000	–	0.024	–	100.000	–		**0md3 l**	–	0.149	0.021	–	–	0.365	0.020	–	–
	**0md3 r**	–	0.061	–	100.000	–	0.028	–	100.000	–		**0md3 r**	–	0.228	0.009	–	–	0.429	0.056	–	–
***Blattella germanica***	**0md1 left**	Complete	0.240	–	100.000	–	0.082	–	100.000	–	***Salganea* sp.**	**0md1 left**	Complete	2.250	–	100.000	–	0.738	–	100.000	–
		a_l	0.078	–	32.494	–	0.021	–	25.419	–			a_l	0.873	–	38.776	–	0.192	–	26.023	–
		b_l	0.052	–	21.772	–	0.020	–	24.116	–			b_l	0.378	–	16.795	–	0.122	–	16.582	–
		c_l	0.032	–	13.403	–	0.014	–	16.843	–			c_l	0.341	–	15.136	–	0.151	–	20.437	–
		d_l	0.007	–	2.746	–	0.003	–	3.485	–			d_l	0.275	–	12.211	–	0.102	–	13.757	–
		e_l	0.025	–	10.396	–	0.011	–	13.567	–			e_l	0.164	–	7.270	–	0.092	–	12.469	–
		f_l	0.030	–	12.324	–	0.007	–	7.959	–			f_l	0.028	–	1.265	–	0.015	–	2.039	–
		g_l	0.016	–	6.865	–	0.007	–	8.612	–			g_l	0.159	–	7.051	–	0.047	–	6.382	–
		h_l	–	–	–	–	–	–	–	–			h_l	0.034	–	1.497	–	0.017	–	2.311	–
	**0md1 right**	Complete	0.322	–	100.000	–	0.131	–	100.000	–		**0md1 right**	Complete	2.788	–	100.000	–	1.234	–	100.000	–
		a_r	0.091	–	28.188	–	0.030	–	22.987	–			a_r	0.945	–	33.899	–	0.295	–	23.876	–
		b_r	0.058	–	17.996	–	0.025	–	18.977	–			b_r	0.319	–	11.451	–	0.150	–	12.124	–
		c_r	0.035	–	10.997	–	0.022	–	16.417	–			c_r	0.433	–	15.542	–	0.219	–	17.715	–
		d_r	0.037	–	11.387	–	0.012	–	9.307	–			d_r	0.457	–	16.378	–	0.182	–	14.731	–
		e_r	0.082	–	25.457	–	0.033	–	25.447	–			e_r	0.435	–	15.606	–	0.287	–	23.224	–
		f_r	0.011	–	3.292	–	0.005	–	4.071	–			f_r	0.076	–	2.720	–	0.037	–	2.982	–
		g_r	0.007	–	2.195	–	0.003	–	2.157	–			g_r	0.102	–	3.665	–	0.052	–	4.187	–
		h_r	0.002	–	0.488	–	0.001	–	0.637	–			h_r	0.021	–	0.739	–	0.014	–	1.161	–
	**0md3 l**	–	0.025	–	100.000	–	0.014	–	100.000	–		**0md3 l**	–	0.111	–	100.000	–	0.066	–	100.000	–
	**0md3 r**	–	0.024	–	100.000	–	0.018	–	100.000	–		**0md3 r**	–	0.124	–	100.000	–	0.069	–	100.000	–

**Note:**

Raw data for the three studied specimens of *P. americana* is provided in Dataset S1. -: not applicable as only one individual was studied.

### Maxillae

**Figure 15:**
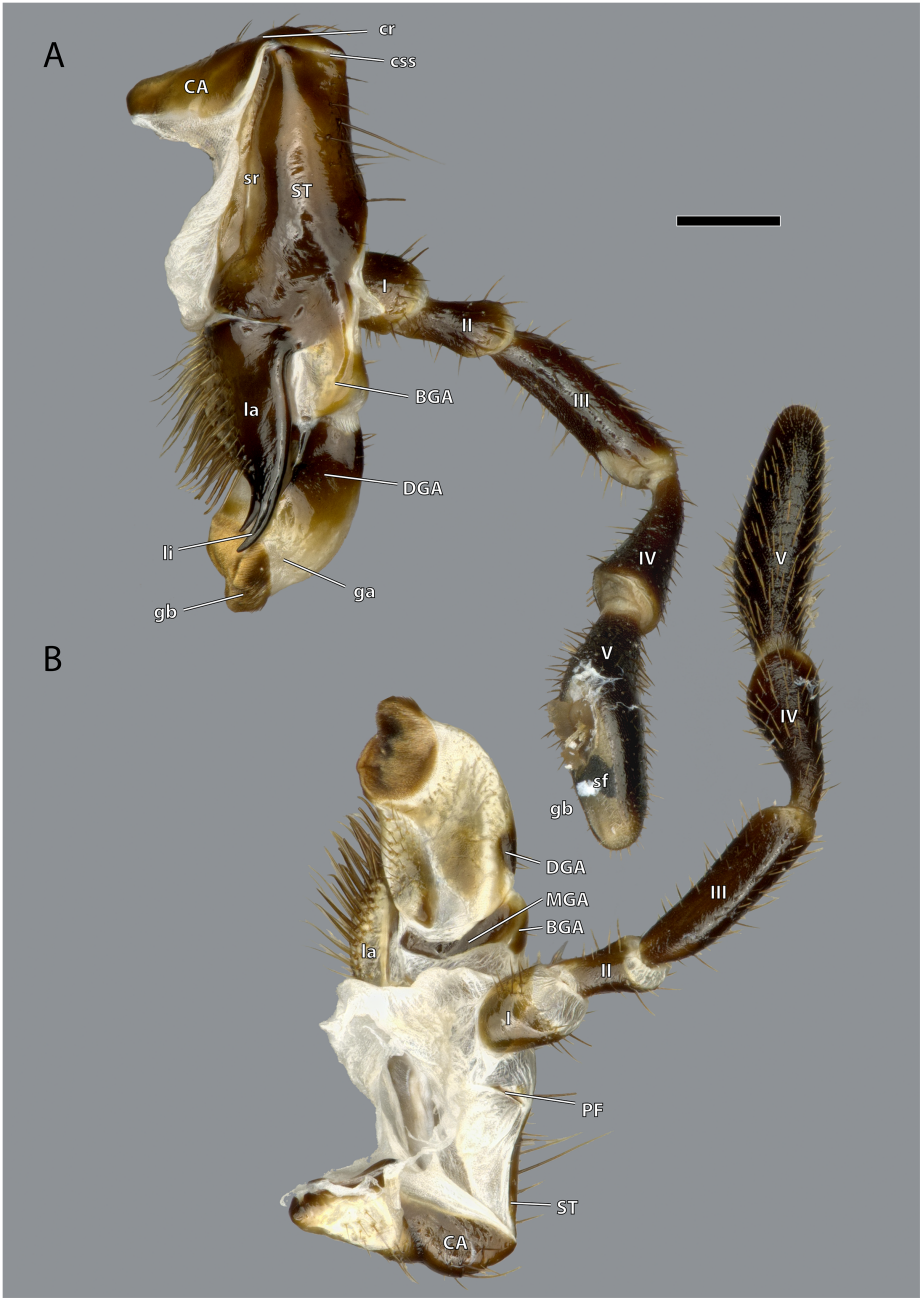
Right maxilla of *Ergaula capucina* ♀. (A) Posterior view; (B) anterior view. Scale bar: 500 µm. Abbreviations: BGA, basigaleal sclerite; CA, cardinal sclerite; cr, cardinal ridge; css, cardino-stipital syndeses; DGA, distogaleal sclerite, ga, galea; gb, galeal brush; la, lacinia; li, lacinial incisivi; MAG, mesogaleal sclerite; PF, palpifer; sf, sensory field of the maxillary palp; sr, stipital ridge; ST, stipital sclerite.

**Figure 16:**
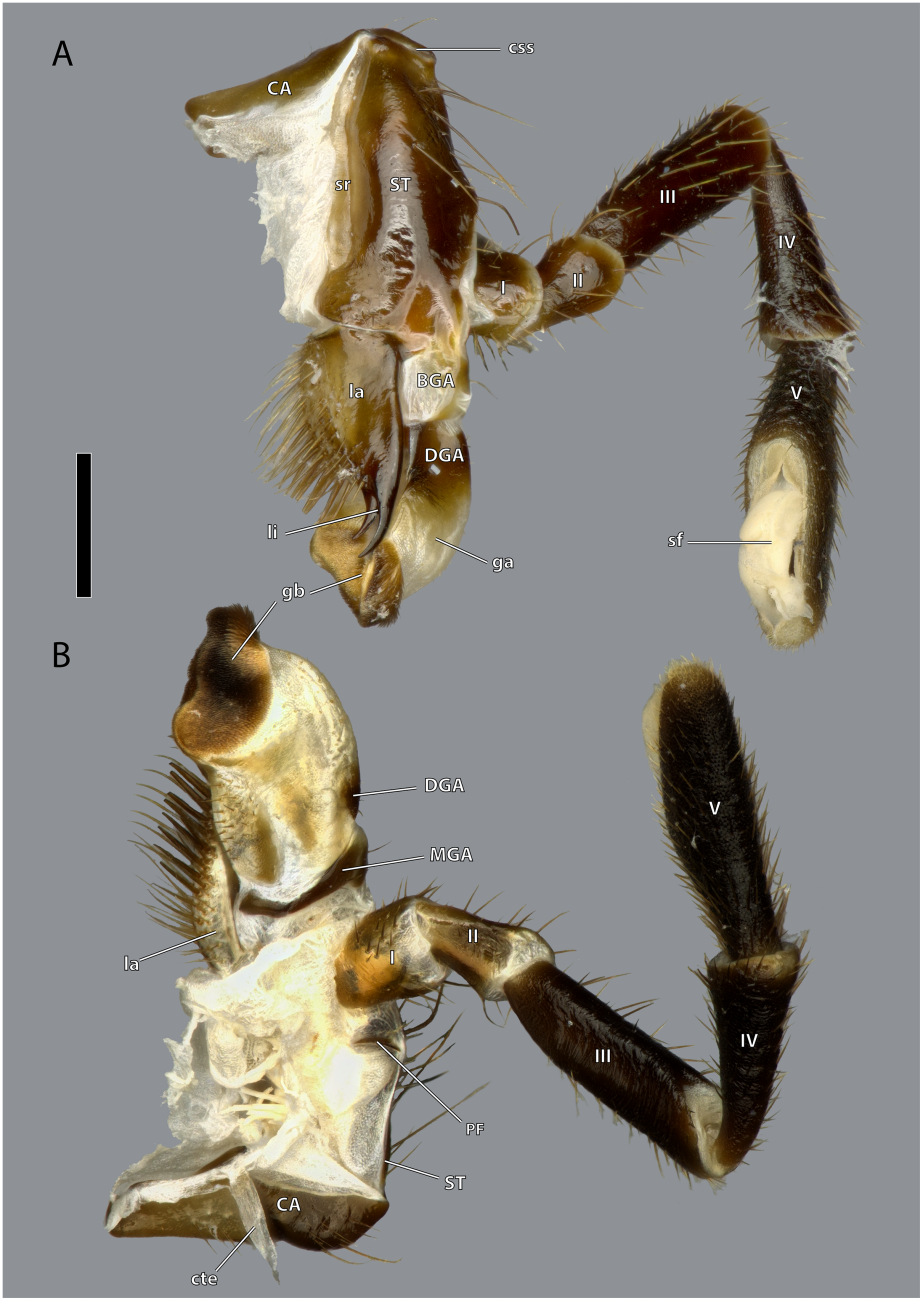
Right maxilla of *Ergaula capucina* ♂. (A) Posterior view; (B) anterior view. Scale bar: 500 µm. Abbreviations: BGA, basigaleal sclerite; CA, cardinal sclerite; cte, cardinal tendon; css, cardino-stipital syndeses; DGA, distogaleal sclerite, ga, galea; gb, galeal brush; la, lacinia; li, lacinial incisivi; MAG, mesogaleal sclerite; PF, palpifer; sf, sensory field of the maxillary palp; sr, stipital ridge; ST, stipital sclerite.

**Figure 17:**
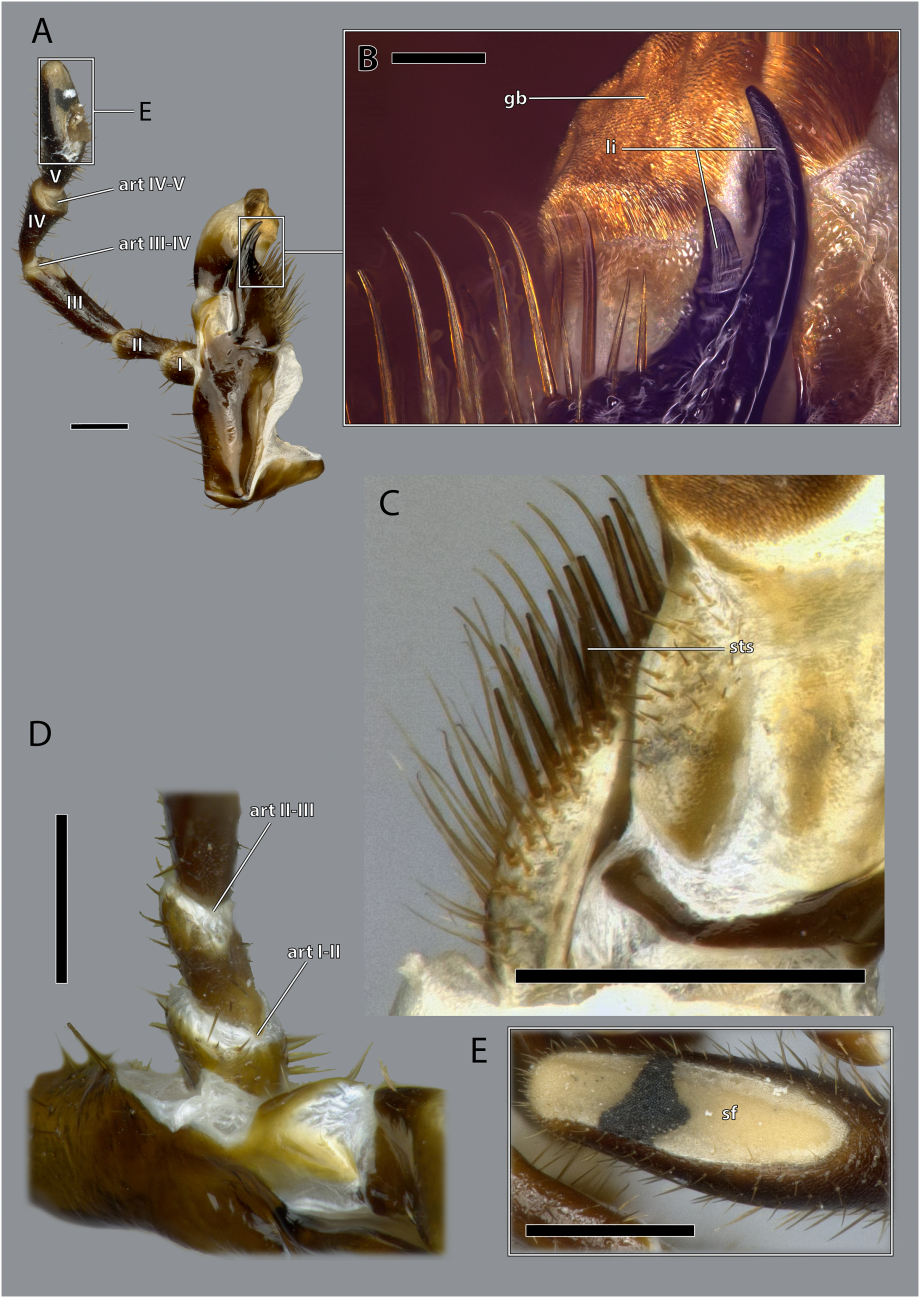
Details on the maxilla of *Ergaula capucina* ♀. (A) Posterior view after maceration; (B) details of the lacinia in posterior view; (C) details of the lacinia in anterior view; (D) lateral view of the articulation of the palp; (E) tip of the palp in posterior view, after maceration. Scale bars: (A), (C)–(E) 500µm; (B) 250µm. Abbreviations: art I-II, mesal articulation of palpomere I and II; art II–III, mesal articulation of palpomere II and III; art III-IV, mesal articulation of palpomere III and IV; art IV–V, mesal articulation of palpomere IV and V; gb, galeal brush; li, lacinial incisivi; sf, sensory field of the maxillary palp; sts, strong setae.

**Figure 18:**
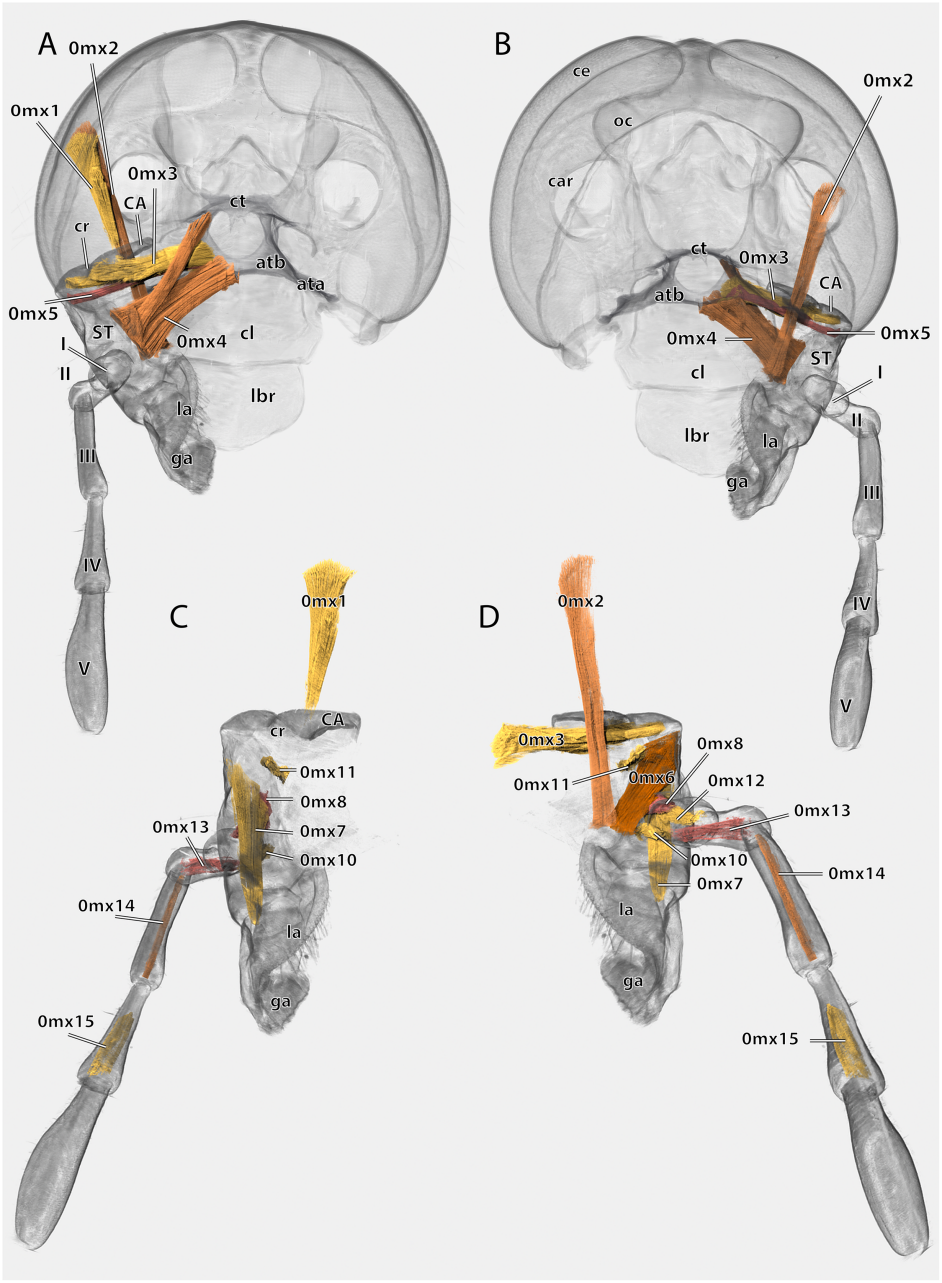
Volume render of the left maxilla including musculature of *Ergaula capucina* ♂. (A) Posterior view, head capsule rendered transparent; (B) frontal view, head capsule rendered transparent; (C) posterior view, cuticle rendered transparent; (D) frontal view, cuticle rendered transparent. Abbreviations: ata, anterior tentorial arm; atb, anterior tentorial bridge; CA, cardinal sclerite; car, circumantennal ridge; ce, compound eye; cl, clypeus; cr, cardinal ridge; ct, corpotentorium; ga, galea; la, lacinia; lbr, labrum; ST, stipital sclerite.

The maxillae (Figs. 15–18) have a strongly sclerotized outer (posterior and lateral) area but are membranous on the anterior side. They articulate with the head capsule along the hypostomal ridge.

The cardo is an elongate and transversely orientated sclerite (CA, Figs. 2, 15, 16, 18) that is covered with a few setae. It is mesally separated into a mesal and lateral half by the cardinal ridge (cr, Figs. 14, 17). On the mesal part, the tendon of M. craniocardinalis (0mx1) (cte, Fig. 15) attaches.

The stipital sclerite (ST, Figs. 2, 3, 15, 16, 18) articulates mesally with the cardinal sclerite. Except this articulation the two sclerites are distinctly separated *via* a membrane (css, Figs. 15, 16). Mesally on the posterior surface of the stipes runs the stipital ridge (sr, Figs. 14, 15) from the cardo-stipital articulation towards the base of the lacinia. It serves as attachment for M. tentoriocardinalis (0mx3). The lateral area of the stipital sclerite is covered with a few long setae. Approximately on the mid-level of the stipital sclerite, the palpifer (PF, Figs. 15, 16) lies embedded in the membranous anterior surface of the maxilla. It is a small, half-moon-shaped sclerite directly basally of the base of the palp. The maxillary palp (mxp, Fig. 2, 3) is five-segmented and articulates with the stipes and the palpifer. Palpomere I is slightly broader than wide, palpomere II broadens distally and is approximately twice as long as its base is broad. They are only sparsely covered with setae. Palpomeres III–V are strongly elongated and densely covered with setae. The palpomeres get increasingly sclerotized from proximal to distal. They are separated by membranes and articulate *via* distinct joints (art I–II, art II–III, art III–IV, art IV–V, Fig. 17). A membranous and oval sensory field (sf, Figs. 2, 3, 15–17) is located on the mesal surface of the distal two-thirds of palpomere V.

The galea (ga, Figs. 3, 15, 16, 18) is a mostly membranes structures with three embedded sclerites: the basi- (BGA, Figs. 15, 16), disto- (DGA, Figs. 15, 16) and the mesogaleal sclerite (MGA, Figs. 15, 16). The basigaleal sclerite lies on the postero-proximal surface of the galea and is partly fused to the stipes. Laterally it goes around the galea and reaches up to the anterior surface. Mesally on the anterior surface the mesogaleal sclerite attaches. It is a long and slender sclerite with a clear border to the surrounding membrane. Meso-distally of the sclerite but distinctly separated lies an oval and slightly sclerotized field with mesally directed setae. The proximal posterior surface is covered by the distogaleal sclerite. In its proximal half it is strongly sclerotized but the sclerotization decreases distally. The distal tip is formed by the galeal brush (gb, Figs. 15–17) that is covered by dense setae.

The lacinia (la, Figs. 15, 16, 18) is positioned postero-mesally of the galea. It contains a single sclerite that is synscleric with the stipital sclerite mesally on the posterior surface but otherwise separated by a thin membranous stripe. The anterio- and postero-mesal surface of the lacinia is covered by several rows of long mesally directed and distally narrowing setae. Mesally on the anterior surface–embedded in between the other setae- lies a row of shorter and stronger setae (sts, Fig. 17) which are more blunt distally. Distally, the lacinia narrows to two strongly sclerotized lacinial incisivi, of which the distal one is stronger and longer (li, Figs. 15–17); a lacinula is absent.

Musculature (Fig. 18): M. craniocardinalis (0mx1)–O: in two bundles, bundle one on the lateral vertex, directly laterally of M. craniomandibularis internus, bundle two on the lateral postgena; I: with a long tendon on the distal tip of the cardinal apodeme. M. craniolacinialis (0mx2)–O: lateral vertex; I: anterior lacinial margin, together with M. stipitolacinialis. M. tentoriocardinalis (0mx3)–O: laterally on the anterior tentorial bridge; I: along the entire posterior inner wall of the cardo on both sides of the cardinal ridge. M. tentoriostipitalis anterior (0mx4)–O: mesally on the anterior tentorial bridge including the mesal ridge, one bundle laterally on the corpotentorium; I: along the entire mesal wall of the stipes including the stipital ridge. M. tentoriostipitalis posterior (0mx5)–O: anteriorly on the anterior tentorial bridge, in between M. tentoriocardinalis and M. tentoriostipitalis anterior; I: dorsally on the inner stipital wall near the stipito-cardinal border, in between the distal parts of M. stipitolacinialis. M. stipitolacinialis (0mx6)–O: with two bundles on the lateral inner stipital wall and with one bundle on the posterior-mesal inner stipital wall; I: anterior lacinial margin, together with M. craniolacinialis. M. stipitogalealis (0mx7)–O: dorsally on the inner stipital wall, directly ventrally of the insertion of M. tentoriostipitalis posterior; I: baso-laterally on the distogalea. M. stipitopalpalis externus (0mx8)–O: dorsally on the lateral wall of the inner stipital ridge; I: dorsally on the base of the first maxillary palpomere. M. stipitopalpalis internus (0mx10)–O: ventro-laterally on the stipital ridge; ventrally on the base of the first palpomere. M. palpopalpalis maxillae primus (0mx12)–O: basally on the anterior wall of the first palpomere; I: meso-basally on the second palpomere. M. palpopalpalis maxillae secundus (0mx13)–O: basally on the anterior wall of the first palpomere; I: latero-basally on the third palpomere. M. palpopalpalis maxillae tertius (0mx14)–O: basally on the anterior wall of the third palpomere; I: meso-basally on the fourth palpomere. M. palpopalpalis maxillae quartus (0mx15)–O: basally on the anterior wall of the fourth palpomere; I: meso-basally on the fifth palpomere.

### Labium

**Figure 19:**
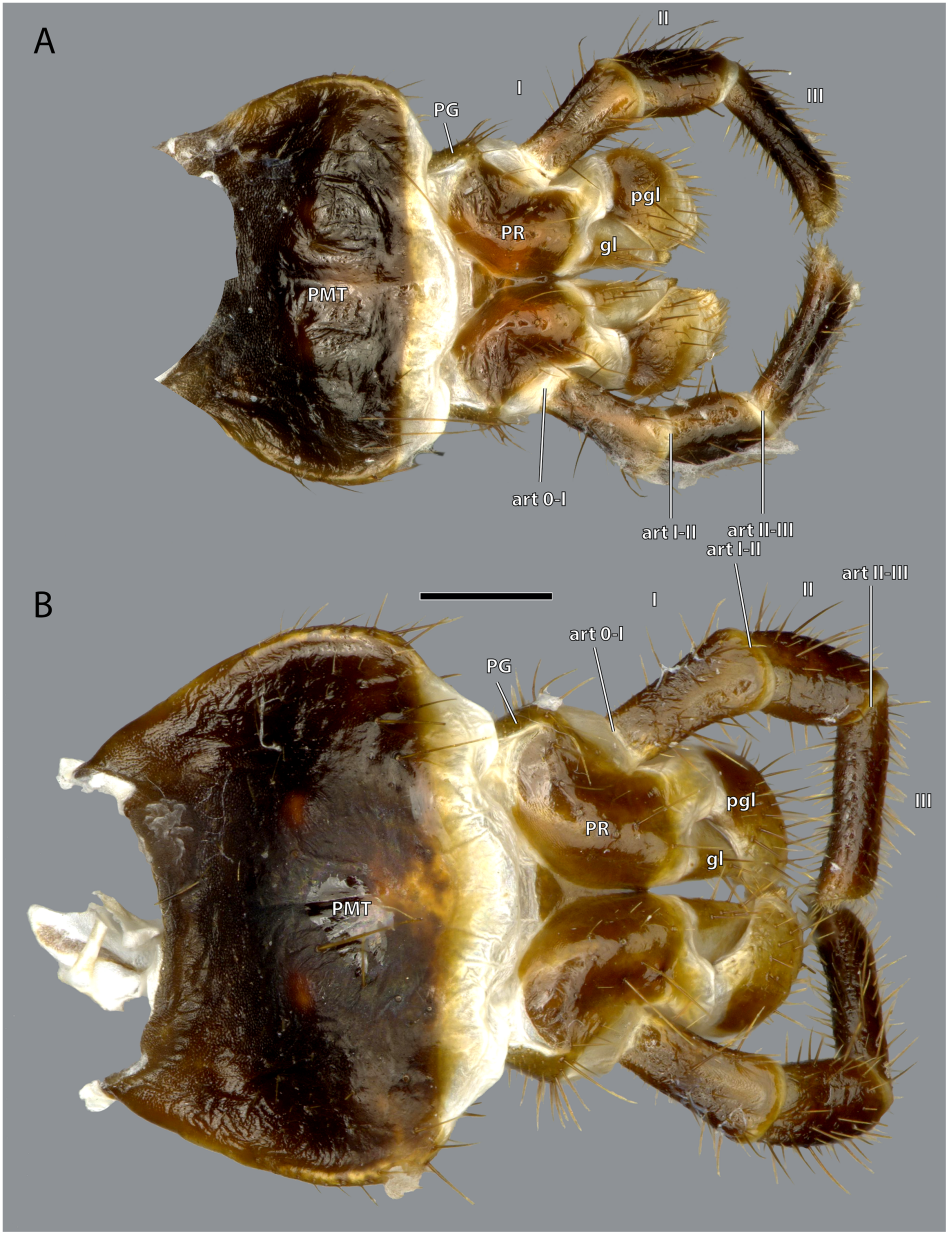
Posterior view of the labium of *Ergaula capucina*. (A) ♂; (B) ♀. Scale bar: 500 µm. Abbreviations: art 0–I, lateral articulation between praemental sclerite and palpomere I; art I–II, posterior articulation of palpomere I and II; art II-III, lateral articulation of palpomere II and III; gl, glossa; PG, palpiger; pgl, paraglossa; PMT, postmental sclerite; PR, praemental sclerite.

**Figure 20:**
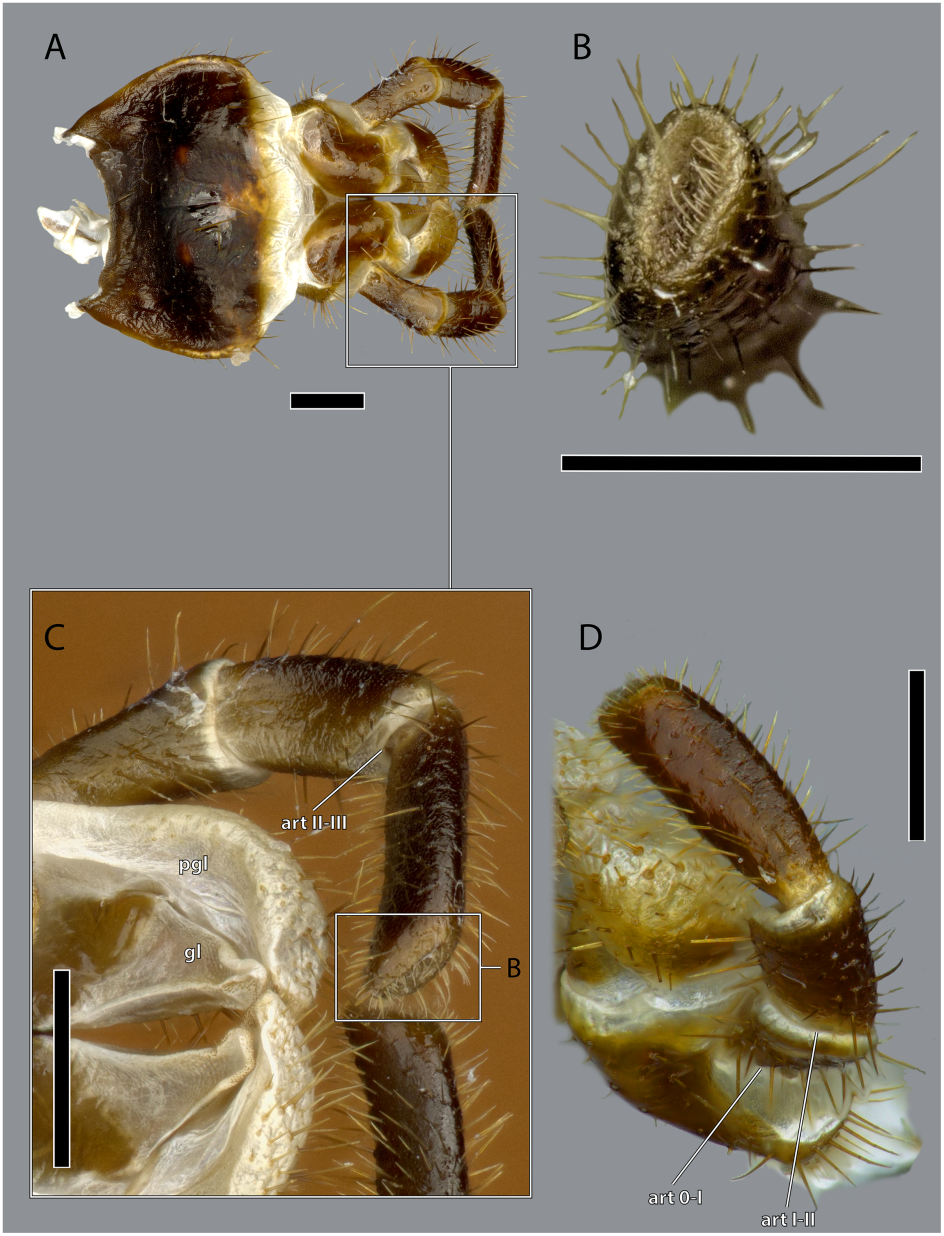
Details on the labium of *Ergaula capucina*. (A) Anterior view; (B) medial view of the tip of the labial palp; (C) posterior view of the distal labium; (D) latero-ventral view of the left labial palp. Scale bars: 500 µm. Abbreviations: art 0–I, lateral articulation between the praemental sclerite and palpomere I; art I–II, posterior articulation of palpomere I and II; art II–III, posterior articulation of palpomere II and III; gl, glossa; pgl, paraglossa.

The labium is composed of an unpaired postmental sclerite (PMT, Figs. 2, 3, 19), an unpaired praemental sclerite (PR, Figs. 2, 3, 19), paired three segmenteal palps (lp, Fig. 2, 3, 10), paired palpigers (PG, Fig. 19), paired glossae (gl, Figs. 3, 19, 20) and paired paraglossae (pgl, Figs. 2, 3, 19, 20).

The postmental sclerite is a strongly sclerotized plate which shows no distinct separation into a submentum and mentum. Several long setae are located around its convex lateral margins while the rest of its surface is only very sparsely covered with setae. The basal edge is concave and connects with the cervical membrane. Basi-laterally, the labium articulates with the head capsule. The praemental sclerite is separated from the postmental one by a broad membrane. It is nearly rectangular with a convex basal margin. Laterally, it has concave embayments that serve as attachment sites for the labial palps. Its surface is sparsely setose. It is mesally divided in its distal half by the praemental cleft. Basally it continues as praemental ridge that divides in the basal part into two lateral ridges, thus forming a triangular area at the praemental base. The sclerotized palpiger lies proximally of the base of the labial palp and laterally of the praementum providing an articulation with the first palpomere. The second articulation of the first palpomere is formed with the praementum. The labial palp is three-segmented and its setation increases apically. The first palpomere is approximately twice as long as wide. The second one is approximately equally sized but slightly thicker. The third palpomere is three times as long as broad. Its distal tip is flattened and densely covered with setae. Palpomeres I and II articulate on the anterior and posterior side while palpomeres II and III have a lateral joint. Two paired lobes are positioned distally of the praementum. The mesal glossae are formed by single and distally pointed sclerites and are weakly covered with setae. The lateral paraglossae are longer and extend mesally over the glossae. They are densely covered with setae. Distally the paraglossae are membranous and densely coated with small setae or microtrichia.

**Figure 21:**
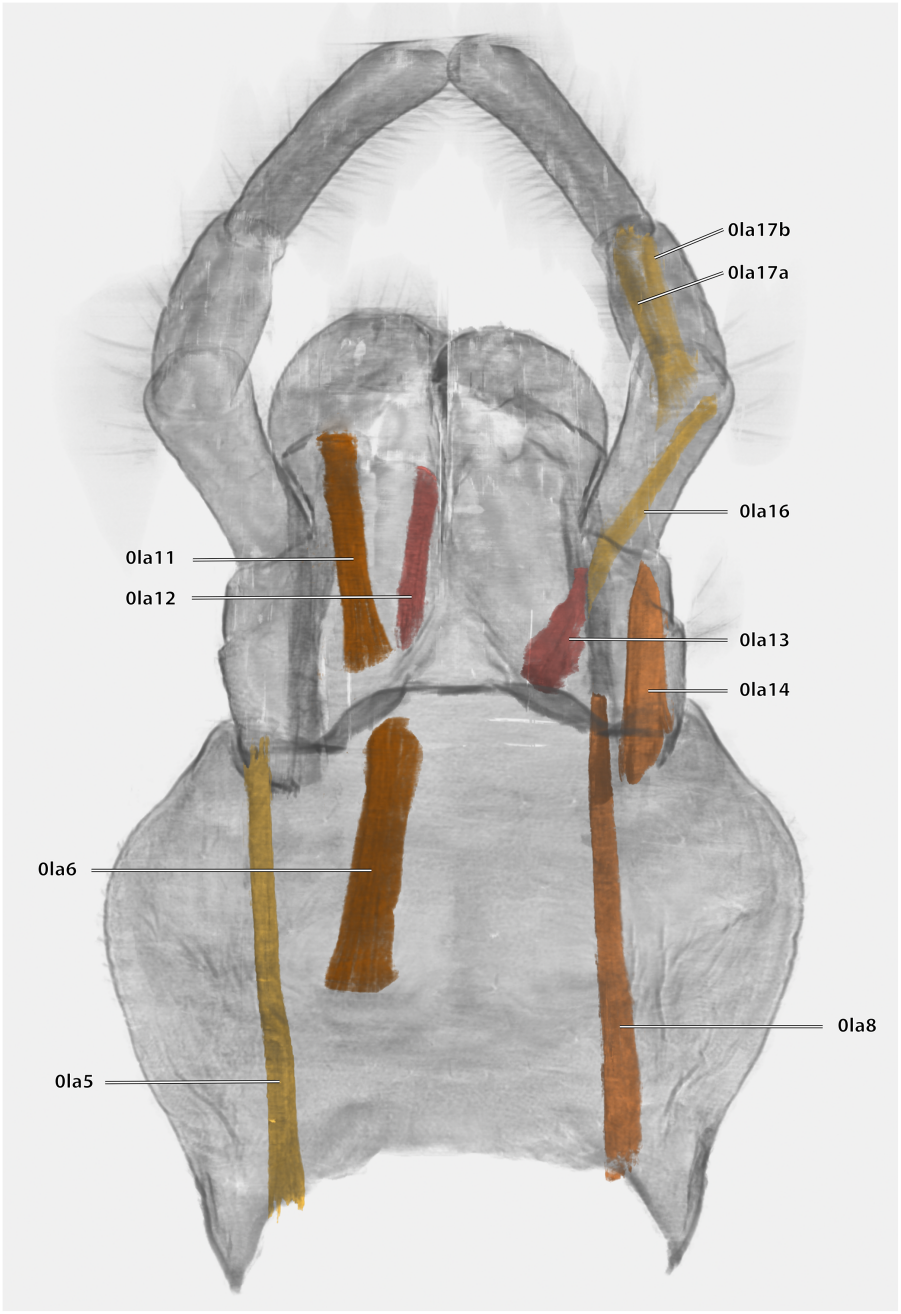
Volume render of the labial musculature of *Ergaula capucina* ♂ in posterior view. Cuticle rendered transparent

Musculature (Fig. 21): M. tentoriopraementalis inferior (0la5)–O: mesally on the anterior part of the trabeculae tentorii; I: laterally at the base of the praementum. M. tentoriopraementalis superior (0la6)–O: anteriorly on the trabeculae tentoria, directly laterally of M. tentoriopraementalis inferior; I: laterally on the basal praementum. M. submentopraementalis (0la8)–O: mesally on the postmentum; I: meso-basally on the praementum. M. praementoparaglossalis (0la11)–O: inner praemental wall, directly laterally of M. praementoglossalis; I: baso-mesal edge of the paraglossa. M. praementoglossalis (0la12)–O: inner praemental wall, laterally of the end of the praemental cleft, directly laterally of M. praementoparaglossalis; I: baso-mesal edge of the glossa. M. praementopalpalis internus (0la13)–O: baso-laterally on the praementum; ventro-basally on the first labial palpomere. M. praementopalpalis externus (0la14)–O: anterio-lateral base of praementum; I: dorso-basal edge of first labial palpomere. M. palpopalpalis labii primus (0la16)–O: anterio-lateral base of praementum, directly ventrally of M. praementopalpalis internus; I: lateral base of the second palpomere. M. palpopalpalis labii secundus (0la17)–O: meso-basal wall of second palpomere; I: in two bundles, one mesally and one laterally in the base of the third palpomere.

### Hypopharynx

**Figure 22:**
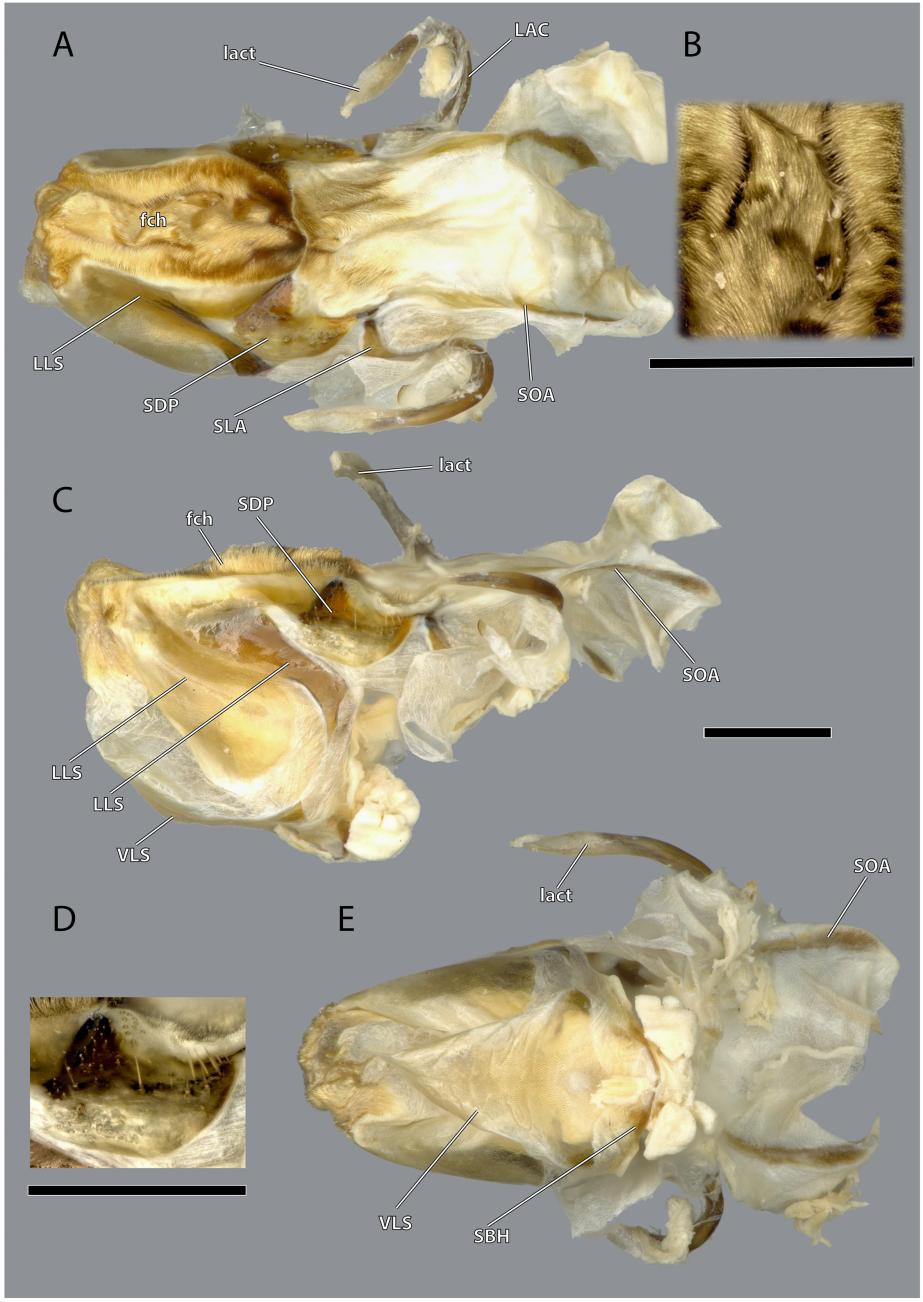
Hypopharynx of *Ergaula capucina* ♀. (A) Frontal view; (B) details of the Microtrichia field in frontal view; (C) lateral view; (D) details of the hypopharyngeal sclerite in antero-mesal view; (E) posterior view. Scale bars: 500µm. Abbreviations: fch, field of microtrichia on the dorsal and distal sclerites of the hypopharynx; LAC, linguactual sclerite; lact, linguactual apodem; LLS, lateral lingual sclerite of the hypopharynx; SBH, arm at the base of the ventral lingual sclerites; SDP, platelike distal part of the hypopharyngeal suspensorium; SLA, loral arm of the hypopharyngeal suspensorium; SOA, oral arm of the hypopharyngeal suspensorium; VLS, ventral lingual sclerite of the hypopharyngeal suspensorium.

The hypopharynx of *Ergaula* is a membranous tongue-like structure with several embedded sclerites. Its apical, anterior surface and parts of the lateral surface are covered with long microtrichia (fch, Fig. 22). It is divided into a distal lingual and a basal suspensorial part by a clear line in these microtrichia. The lateral lingual sclerite (LLS, Fig. 22) is embedded in the lateral part of the distal hypopharynx. It is divided into a weakly sclerotized anterior and a stronger sclerotized posterior part. Dorsally this sclerite reaches towards the field of microtrichia. Ventro-posteriorly it continues towards the base of the hypopharynx where it fuses with the arm of the ventral lingual sclerite (SBH, Fig. 22), an arm-like projection that frames the ending of the salivary duct. Dorso-posteriorly, the lateral lingual sclerite fuses with the sickle-shaped plate-like distal part of the hypopharyngeal suspensorium (SDP, Fig. 22) that is otherwise separated by membranes. The oral arm of the suspensorium (SOA, Fig. 22) continues posterio-dorsally from the plate-like distal part of hypopharyngeal suspensorium and frames the pharynx. At the base of the oral arm, the oral arm of the suspensorium (SLA, Fig. 22) continues ventro-posteriorly. The linguactual sclerite (LAC, Fig. 22) continues posteriorly and performs an anterior bend; its apodeme (lact, Figs. 10, 21) continues into the mandible. The ventral side of the hypopharynx is formed by the ventral lingual sclerite (VLS, Fig. 22).

Musculature (Fig. 10): M. frontooralis (0hy1)–O: frons, directly laterally of M. frontoepipharyngalis; I: distal tip of the oral arm of the hypopharynx. M. tentoriooralis (0hy2)–O: subgena, in between the antennal base and the anterior mandibular articulation; I: laterally on the oral arm of the hypopharynx, proximally of the insertion of M. frontooralis. M. tentoriohypopharyngalis (0hy3)–O: anteriorly on the trabeculae tentorii, laterally of M. tentoriopraementalis superior; I: dorsal side of the base of the ventral lingual sclerite. M. tentoriosuspensorialis (0hy5)–O: oesotendons, together with M. tentoriobuccalis anterior and posterior; I: mesally on the hypopharyngeal membrane, slightly ventrally of the anatomical mouth opening. M. praementosalivarialis anterior (0hy7)–O: posterior wall of the praementum, mesally of the articulation of the labial palp; I: proximally on the ventral side of the basal hypopharyngeal apodeme. M. praementosalivarialis posterior (0hy8)–O: anterio-lateral wall of the base of the praementum; slightly mesally of M. praementopalpalis externus. *M. oralis* transversalis (0hy9)–O: oral arm of the hypopharynx; I: oral arm of the opposite side, fibers run on both sides of the pharynx. M. loroloralis (0hy10)–O: mesal wall of the lateral hypopharyngeal sclerite; I: mesal wall of the lateral hypopharyngeal sclerite. M. hypopharyngosalivarialis (0hy12)–O: anteriorly on the loral sclerite; I: along the entire membranous floor of the hypopharynx.

### Pharynx

The pharynx (ph, Figs. 5, 10) is oval in cross section and longitudinally folded to increase the inner surface. The functional mouth opening is located anteriorly of the frontal ganglion and is flanked on both sides by the oral arms of the hypopharynx.

Musculature (Fig. 10): M. clypeobuccalis (0bu1)–O: clypeus, directly dorsally of M. clypeopalatalis; I: anterior pharynx, in between the bundles of *M. oralis* transversalis, directly before the functional mouth opening. M. frontobuccalis anterior (0bu2)–O: mesally on the frons; I: anterior pharynx, directly dorsally of the frontal connective. M. frontobuccalis posterior (0bu3)–O: mesally on the frons, directly laterally of the origin of M. frontolabralis; I: anterior surface of pharynx, dorsally of the insertion of M. frontobuccalis anterior. M. tentoriobuccalis lateralis posterior (0bu4)–O: corpotentorium; I: lateral pharyngeal wall, next to M. tentoriopharyngalis. M. tentoriobuccalis anterior (0bu5) and M. tentoriobuccalis posterior (0bu6, not figured)–O: oesotendons, together with M. tentoriosuspensorialis; I: along the posterior wall of the pharynx, slightly ventrally of the insertion of M. frontobuccalis anterior. M. tentoriobuccalis lateralis anterior (0bu7)–O: subgena, in between the antennal base and the anterior mandibular articulation, dorsally of M. tentoriooralis. M. verticopharyngalis (0ph1)–O: in two bundles on the vertex, in between the bundles of M. craniomandibularis internus; I: two bundles on the anterior surface of the pharynx, first bundle directly dorsally of the brain, second one further dorsally. M. tentoriopharyngalis (0ph2)–O: anteriorly on the border between the posterior tentorial arms and the corpotentorium; I: posterior wall of the pharynx, together with the insertion of M. verticopharyngalis. M. postoccipitopharyngealis (0ph3)–O: postoccipital ridge laterally of the foramen occipitale; I: along the lateral pharyngeal wall, close the point where the pharynx leaves the head capsule. M. anularis stomodaei (0st1, not figured)–ring muscle along the cephalic pharynx. *M. longitudinalis* stomodaei (0st2, not figured)–longitudinal muscle layer along the cephalic pharynx.

### Central nervous system

The supraoesophageal ganglion (sog, Fig. 5) is prominent. Proto-, deutero-, and tritocerebrum are not externally distinguishable. Frontally, nerves run towards the lateral ocelli. The nerve associated with the central ocellus is completely missing. The antennal nerve (ann, Fig. 5) and the optical lobes are well developed. The frontal connectives (frc, Fig. 5) originate ventrally on the supraoesopharyngeal ganglion and run around the pharynx where they connect with the frontal ganglion (fg, Fig. 5). The nervus recurrens (ner, Fig. 5) runs dorsally from the frontal ganglion along the anterior side of the pharynx below the brain towards the corpora allata. The supraoesophageal ganglion is connected with the smaller suboesophageal ganglion (sug, Fig. 5) *via* circumoesophageal connectives. The tritocerebral commissure runs ventrally around the pharynx.

## Discussion

### Morphological aspects

*Ergaula capucina* shows a strong sexual dimorphism in the head capsule. Males have a distinctly smaller head capsule and considerably larger compound eyes (Figs. 2–4). The latter reach towards the subgenal ridge in males while in females they end further dorsally of them. Similar sexual dimorphisms are found among other cockroaches and are most pronounced in species with actively flying males and wingless females (Bell, Roth & Nalepa, 2007). Anisyutkin (2014) speculated that this dimorphism is a result of retardational paedomorphosis as the females resemble the juvenile form. Other cephalic sense organs such as the antennae (Lambin, 1973) and maxillary palps (Bland, Slaney & Weinstein, 1998) are affected by sexual dimorphisms as well. Other parts of the body such as the abdomen, the shape of the pronotum or the presence of wings are also affected by sex-related differences. Extreme examples of sexual dimorphisms in cockroaches include the genera *Escala* and *Robshelfordia* (Bell, Roth & Nalepa, 2007; Roth, 1991). Nevertheless, sex-related differences are commonly found among insects (Stillwell et al., 2010).

The tentorium of *Ergaula* follows the general blattodean pattern of having twisted and fused anterior tentorial arms (“perforate tentorium”), oesotendons, and short, stout posterior tentorial arms (Klass & Eulitz, 2007). However, it differs from all other cockroach species whose tentorium has been described in not having dorsal tentorial arms. Their absence also has consequences for the musculature. Muscles which usually attach to these arms are relocated. One bundle of both M. tentorioscapalis anterior and posterior normally attaches to the dorsal tentorial arms (Wipfler et al., 2016) but these bundles are lacking in *Ergaula*. The origination of M. tentoriobuccalis lateralis posterior is normally on the tentorial arms, but in *Ergaula* it originates on the corpotentorium. These changes in origin have no effect on the point of insertion in any of the affected muscles.

The blattodean epipharynx, including that of *Ergaula*, follows a general scheme of a mesal epipharyngeal brush being laterally framed by four to 50 epipharyngeal spikes, tormae, and an epipharyngeal suspensorium (Zhuzhikov, 2007). Additionally, *Ergaula* has an epipharyngeal gland or frontal body that is located dorsally of the tormae in the epipharyngeal wall. It is connected *via* an epipharyngeal ductus to a gland opening at the distal margin of the epipharynx. Similar structures are also reported for other genera of Corydiidae [*Arenivaga investigata* (O’Donnell, 1977a, 1977b, 1981) and *Polyphaga aegytiaca* (Zhuzhikov, 2007)]. This gland is a part of an apparatus that absorbs atmospheric water-vapor and also includes protrudable bladders on the hypopharynx (O’Donnell, 1977a, 1977b). When the relative humidity reaches a certain threshold (82.5% in *Arenivaga investigata*; Edney, 1966), bladders on the lateral hypopharynx are inflated and protruded. There is no description of which exact part of the hypopharynx belongs to the bladder but it seems likely to be the lateral lingual sclerite as it forms the lateral wall of the distal hypopharynx. The cuticle of this bladder absorbs water-vapor from the air while the secretion of the epipharyngeal gland that is channeled to the bladder *via* the epipharyngeal duct may help in releasing this stored water into the mouth cavity (O’Donnell, 1982). This gland is operated by a prominent M. clypeopalatalis (0ci1), which is also found in *Ergaula* (Fig. 10). Corydiid cockroaches are known to inhabit xeric areas (Bell, Roth & Nalepa, 2007) and show a wide array of different adaptations towards the dry climate including a roundish body shape that provides a low volume to body surface ratio (Evangelista et al., 2019). The unique vapour-absorption apparatus also belongs to those adaptations. This inflation and protrusion of the hypopharyngeal bladder was directly observed in *Arenivaga investigata* (O’Donnell, 1977a, 1977b, 1981), *Heterogamisca chopardi* (Grandcolas, 1994), and *Polyphaga aegyptiaca* (Vannier & Ghabbour, 1983). Grandcolas (1994) also tested for this behavior in *Ergaula capensis*, *Therea petiveriana*, and *Polyphaga aegyptiaca* with negative results. However, the latter species has homologous epipharyngeal glands (Zhuzhikov, 2007) and was shown to be capable of absorbing water by another study (Vannier & Ghabbour, 1983), which might imply that this behavior might be triggered by special conditions. Based on these facts and the assumption that the gland and the inflatable bladder form a single functional unit that cannot properly function with some parts missing (O’Donnell, 1977a, 1977b, 1982), it is likely that all corydiid cockroaches with an epipharyngeal gland, including *Ergaula*, have the potential to absorb atmospheric water-vapour. The only Corydiidae species to be examined, thus far, are in Corydiinae. The state of the other clades is unknown.

*Ergaula* has a small sclerotized plate on the posterior side of the mandible near the articulation with the tendon of M. craniomandibularis internus. A comparable structure is not found in *Periplaneta americana* (Wipfler et al., 2016) but reported (as basatendon) for some not further specified cockroaches by Zhuzhikov (2007). According to this author, it serves as additional joint between the mandible and the tendon.

The most interesting morphological feature on the maxillae of *Ergaula* is the simultaneous presence of strong, blunt, and shorter setae and longer, weaker, and tipped setae in combination with the absence of a lacinula. The latter is a small structure positioned directly at the base of the lacinial incisivi (Zhuzhikov, 2007). Lacinulae are found in all cockroaches with the exception of Corydiidae with a wide range of shape (Zhuzhikov, 2007). At the same time, *Ergaula* is the only studied species of cockroaches with more massive and blunt setae in the mesal lacinial margin. It is possible that these stronger setae are homologues to the lacinula of other cockroaches. Outside of cockroaches, lacinula-like structures are also found in orthopterans and stoneflies (Crampton, 1923; Eidmann, 1923). It was speculated that these structures are homologues to those found in Collembola, Archaeognatha, and Zygentoma (Crampton, 1923) and to the dentisetae of mayflies and dragonflies (Blanke et al., 2012; Wipfler et al., 2016). Currently we lack data to interpret these structures in an evolutionary context.

*Ergaula* lacks a stipital disc at the base of the lacinia, which is present in *Periplaneta* (Wipfler et al., 2016). It is likely that this loss is associated with the reduction of M. stipitopalpalis medialis (0mx9) that attaches at this point. However, only these two species have been studied with regard to this feature, so no sound evaluation of this character is possible.

### Functional considerations

The general construction of the mandibular apparatus of *Ergaula* resembles those of other cockroaches and most other polyneopteran insects (Yuasa, 1920; Wipfler et al., 2011, 2012, 2016). The mandible is a single sclerite that is connected to the head capsule *via* two joints (dicondylic) and thus can only swing in a single plane. Its mesal side is covered by incisivi and a molar region. Additionally, cockroaches, termites and mantids (together Dictyoptera) share a membranous postmola at the base of the mesal side (Vishnoi, 1956; Wipfler et al., 2012, 2016). The movement of the mandible is operated by the same set of muscles as in all polyneopteran insects. An important characteristic of the mandibular apparatus is the mechanical advantage (MA), *i.e.*, the force transmission ratio of the mandible. The MA is defined as the quotient of the inner lever (*i.e.*, the distance between the attachment of the mandibular adductor and the rotation axis of the mandible) and the outer lever (*i.e.*, the distance between the rotation axis of the mandible and the point of interest on the mandibular mesal surface). Lower values correspond to lower transmission of muscle force to the respective point. Table 2 lists the mechanical advantage of the different incisivi of the mandibles of *Ergaula*. The right and left one do not differ in terms of the most distal one (0.37). However, the most proximal ones show distinct differences (0.45 to 0.52), which is correlated to the fact that the left one is distinctly more proximal than the right. Similar values (ranging between 0.37 and 0.47) have been measured for *Periplaneta americana* (Weihmann et al., 2015a) but also for carnivorous beetles (0.35–0.59); (Wheater & Evans, 1989). Seemingly, there is no direct correlation between diet and mechanical advantage (Weihmann et al., 2015a).

The primary mandibular adductor muscle M. craniomandibularis internus (0md1) is attached to the mandible *via* a massive tendon. In *Ergaula*, this muscle has eight distinct bundles (Table 2)−a pattern also found in *Periplaneta americana* (Weihmann et al., 2015a), *Salganea rossi*, and *Blattella germanica* (Table 3). Another shared feature of all these cockroaches is the asymmetry of this muscle. In all four species, the right M. craniomandibularis internus (0md1) has more volume than the left one and parts of the right muscle enter the left half of the body (Table 3). This is also reflected in the effective cross section area that is distinctly larger (Table 3). The most extreme case of this right/left asymmetry is bundle “h” in *Blattella germanica*. The muscle that inserts on the left body half changed its origin to the right side. Strong differences are also found in the volume and effective cross section of the individual bundles of one body side (Table 3). For example, bundle “a” is the largest one in all studied species. Similar discrepancies between the right and the left side of M. craniomandibularis internus (0md1) are not found in termites (Vishnoi, 1962). The close relatives of Blattodea, mantises (Levereault, 1938; Wipfler et al., 2012) and other polyneopteran insects (Friedemann et al., 2012; Matsumura et al., 2015; Neubert et al., 2017; Wipfler et al., 2011), also do not show this right/left asymmetry in the mandibular adductor. We currently have no explanation for this asymmetry since, from a functional point of view, each muscle must generate equal forces to keep the mandibular apparatus in a functional equilibrium. Although the present study provides the first detailed measurements of the volume and cross section of the bundles of the right and left side of M. craniomandibularis internus, it is only the first step towards a better understanding of the nature of this asymmetry. Further analyses need to include a broader taxon sampling. The same applies to other characteristics of the mandibular apparatus such as bite forces, bite duration, or opening ranges, which only have been measured for *Periplaneta americana* (Weihmann et al., 2015b).

### Phylogenetic implications

The present study provides the first detailed examination of the head morphology of a species of Corydioidea. This group was the last of the three major lineages of Blattodea for which a detailed morphological treatment of the head capsule was missing. The filling of this gap thus allows a better evolutionary and phylogenetic interpretation of the blattodean head. Our results of *Ergaula* confirm the “perforate” tentorium, the membranous postmola, and a lacinia that lies in a galeal pouch (Wipfler et al., 2012, 2016) as dictyopteran apomorphies. For Blattodea, we support the following characters as apomorphies: the absence of the median ocellus, a bipartite M. verticopharyngealis (0ph1), and a bipartite M. hypopharyngosalivaris (0hy12). Additionally, it was discussed that oesotendons, which are present in all groups except Tutricablattae (*i.e.*, Cryptocercidae and Isoptera), are potential apomorphies of Blattodea (Klass & Eulitz, 2007; Wipfler et al., 2016). The well resolved genomic phylogeny of Evangelista et al. (2019) confirms that Tutricablattae are nested within Blattoidea and thus oesotendons also belong to the ground plan of Blattodea and are apomorphic for the group.

The absence of a distinct lacinula and the presence of stronger and blunt setae in *Ergaula* are interesting phylogenetic characters. As outlined above, it is possible that these setae in *Ergaula* are homologues to the lacinula of other cockroaches. The construction and variation of these structures might be a useful character to delimit relationships within Blattodea. Additionally, the form found in *Ergaula* resembles the structures in orthopterans and stone flies (Crampton, 1923; Eidmann, 1923) as well as the dentisetae in mayflies and odonates (Blanke et al., 2012). If they are indeed homologues, this would invalidate the presence of dentisetae as a potential argument for the monophyly of Palaeoptera (Blanke et al., 2012, 2013). The detailed comparison of these structures among the different groups should be of high importance.

Recent phylogenetic studies (Bourguignon et al., 2018; Evangelista et al., 2018, 2019) support the monophyly of Corydiinae. The presence of a water-vapor absorption complex including epipharyngeal glands and hypopharyngeal bladder structures (outlined above) could thus be a synapomorphy for Corydiinae but other corydiid taxa (*e.g*., Euthyrrapha, Tivia, Holocompsa) and other Corydiidae *sensu* lato (*e.g*., Nocticola, Latindiinae) should be examined to finally settle this issue.

Other characters, such as the subdivision of the postmentum in submentum and mentum, may correlate with the length-width ratio of this plate rather than with any phylogenetic signal. Zhuzhikov (2007) shows that all species, regardless of their phylogenetic classification, with a width to length ratio higher than 0.65 have an undivided postmentum and all with a lower ratio have a submentum and mentum. This hypothesis should be further tested on a phylogenetic framework.

## Conclusions

Corydioidea was the last of the three major lineages of Blattodea for which a detailed morphological treatment of the head capsule was missing. The present study closes this gap by providing detailed morphological, functional and phylogenetic information for the head of *Ergaula capucinea* and thus allows us to soundly address morphological and phylogenetic aspects of the blattodean ground plan. Blattodea is characterized by various cephalic apomorphies including the reduction of the median ocellus, oesotendons, and bipartite M. verticopharyngealis and M. hypopharyngosalivaris. Another very interesting character in the head of *Ergaula* are the strong and blunt setae that resemble structures found in stoneflies, orthopterans, mayflies and odonatans. If all these structures are indeed homologues, which has to be clarified by future studies, this would invalidate a major morphological argument for monophyletic Palaeoptera. *Ergaula* and other corydiid roaches have a water absorption apparatus comprising the epi- and hypopharynx. This unique device that absorbs atmospherical water into the cuticle should receive stronger scientific attention and should also be studied with respect to transferring its functional principle into a technical way to gain water from high humidity.

From a functional point of view, the present study is the first one to describe an asymmetry between the right and left mandibular adductor of cockroaches with the latter being more massive and also entering the left body half. A similar pattern is not observed in termites, mantids or other closely related insects. This phenomenon can currently not be properly explained and definitely needs further research.

## Supplemental Information

10.7717/peerj.12470/supp-1 Supplemental Information 1 Raw data for the individual measurements for the bundles of the mandibular muscles in *Periplaneta americana*. Specimen numbers (#) are identical to Weihmann et al. 2015.

## Additional Information and Declarations

### Data Availability

The following information was supplied regarding data availability: The CT scan is available at Morphobank: Project 4105: B. Wipfler, F. Triesch, D. Evangelista, T. Weihmann. 2022. Morphological, functional and phylogenetic aspects of the head capsule of the cockroach *Ergaula capucina* (Insecta/Blattodea). PeerJ. (In Press) https://morphobank.org/index.php/Projects/ProjectOverview/project_id/4105.
