# Supplementary material for: Morphological, functional, and phylogenetic aspects of the head capsule of the cockroach Ergaula capucina (Insecta/Blattodea)
Source: PeerJ. 2022 Apr 19;10:e12470. doi: 10.7717/peerj.12470 (PMC9029459; doi:10.7717/peerj.12470)
Supplement: Supplemental Information 1 — Specimen numbers (#) are identical to Weihmann et al. 2015. [file peerj-10-12470-s001.pdf]

Supplementary table 1: Individual measurements for the bundles of the mandibular muscles in *Periplaneta americana.* Specimen numbers (#) are identical to Weihmann et al. 2015

		Area [mm^2]						Area [%]				
		# 3	#4	#12	Average	Standard deviation		# 3	#4	#12	Average	Standard deviation
0md1 left	complete	2,257	1,813	2,225	2,098	0,247		100,000	100,000	100,000	100,000	0,000
	a_l	0,581	0,481	0,535	0,532	0,051		25,763	26,502	24,061	25,442	1,252
	b_l	0,537	0,366	0,574	0,492	0,111		23,776	20,175	25,792	23,248	2,845
	c_l	0,334	0,233	0,393	0,320	0,081		14,820	12,871	17,661	15,117	2,409
	d_l	0,173	0,130	0,160	0,154	0,022		7,654	7,189	7,174	7,339	0,273
	e_l	0,218	0,344	0,223	0,262	0,072		9,639	18,993	10,042	12,892	5,288
	f_l	0,219	0,108	0,148	0,158	0,056		9,698	5,935	6,667	7,433	1,995
	g_l	0,161	0,133	0,168	0,154	0,018		7,138	7,347	7,531	7,339	0,196
	h_l	0,034	0,018	0,024	0,025	0,008		1,511	0,987	1,071	1,190	0,281
0md1 right	complete	2,556	2,310	2,202	2,356	0,181		100,000	100,000	100,000	100,000	0,000
	a_r	0,554	0,597	0,444	0,532	0,079		21,656	25,855	20,163	22,558	2,951
	b_r	0,595	0,480	0,449	0,508	0,077		23,264	20,795	20,394	21,484	1,554
	c_r	0,282	0,375	0,344	0,334	0,048		11,020	16,243	15,639	14,301	2,857
	d_r	0,298	0,231	0,251	0,260	0,034		11,662	10,017	11,395	11,025	0,883
	e_r	0,599	0,427	0,482	0,503	0,088		23,429	18,499	21,884	21,271	2,522
	f_r	0,104	0,087	0,098	0,096	0,008		4,054	3,765	4,459	4,093	0,349
	g_r	0,081	0,052	0,107	0,080	0,027		3,153	2,269	4,840	3,420	1,306
	h_r	0,045	0,059	0,027	0,044	0,016		1,762	2,559	1,227	1,849	0,670
**0md2 left**		0,138	0,135	0,173	0,149	0,021		-	-	-	-	-
**0md 2 right**		0,229	0,237	0,219	0,228	0,009		-	-	-	-	-

		Volume [mm^3]						Volume [%]				
		# 3	#4	#12	Average	Standard deviation		# 3	#4	#12	Average	Standard deviation
0md1 left	complete	3,055	2,156	3,125	2,779	0,540		100,000	100,000	100,000	100,000	0,000
	a_l	0,803	0,626	0,749	0,726	0,091		26,302	29,012	23,969	26,428	2,524
	b_l	0,603	0,398	0,683	0,561	0,147		19,738	18,448	21,868	20,018	1,727
	c_l	0,637	0,306	0,724	0,556	0,221		20,848	14,176	23,169	19,398	4,668
	d_l	0,132	0,115	0,158	0,135	0,022		4,314	5,328	5,053	4,898	0,524
	e_l	0,383	0,406	0,394	0,394	0,011		12,551	18,825	12,592	14,656	3,610
	f_l	0,209	0,130	0,184	0,174	0,040		6,828	6,024	5,898	6,250	0,505
	g_l	0,209	0,141	0,194	0,181	0,035		6,826	6,556	6,209	6,530	0,310
	h_l	0,079	0,035	0,039	0,051	0,024		2,592	1,630	1,242	1,822	0,695
0md1 right	complete	3,959	3,432	3,358	3,583	0,328		100,000	100,000	100,000	100,000	0,000
	a_r	0,887	0,841	0,755	0,828	0,067		22,398	24,516	22,476	23,130	1,201
	b_r	0,714	0,626	0,673	0,671	0,044		18,033	18,244	20,029	18,769	1,097
	c_r	0,702	0,657	0,558	0,639	0,074		17,737	19,147	16,628	17,838	1,262
	d_r	0,324	0,338	0,297	0,320	0,021		8,175	9,849	8,845	8,957	0,843
	e_r	0,971	0,697	0,765	0,811	0,143		24,518	20,299	22,775	22,531	2,120
	f_r	0,179	0,109	0,125	0,138	0,037		4,534	3,165	3,716	3,805	0,689
	g_r	0,100	0,084	0,141	0,108	0,030		2,517	2,438	4,196	3,051	0,993
	h_r	0,083	0,080	0,045	0,069	0,021		2,088	2,342	1,334	1,921	0,524
**0md2 left**		0,389	0,352	0,355	0,365	0,020		-	-	-	-	-
**0md 2 right**		0,481	0,436	0,370	0,429	0,056		-	-	-	-	-
